# Short-Term Outcomes Analysis Comparing Open, Lap-Assisted, Totally Laparoscopic, and Robotic Total Gastrectomy for Gastric Cancer: A Network Meta-Analysis

**DOI:** 10.3390/cancers16193404

**Published:** 2024-10-06

**Authors:** Michele Manara, Alberto Aiolfi, Gianluca Bonitta, Diana Schlanger, Calin Popa, Francesca Lombardo, Livia Manfredini, Antonio Biondi, Luigi Bonavina, Davide Bona

**Affiliations:** 1 I.R.C.C.S. Ospedale Galeazzi–Sant’Ambrogio, Division of General Surgery, Department of Biomedical Science for Health, University of Milan, Via C. Belgioioso, 173, 20157 Milan, Italy; michele.mnra@gmail.com (M.M.); bbonit@icloud.com (G.B.); francesca.lombardo89@gmail.com (F.L.); manfredini.livia@gmail.com (L.M.); davide.bona@unimi.it (D.B.); 2 Surgery Clinic 3, Regional Institute of Gastroenterology and Hepatology “Prof. Dr. Octavian Fodor”, “Iuliu Hațieganul” University of Medicine and Pharmacy, 400394 Cluj-Napoca, Romania; schlanger.diana@yahoo.com (D.S.); calinp2003@yahoo.com (C.P.); 3 G. Rodolico Hospital, Surgical Division, Department of General Surgery and Medical Surgical Specialties, University of Catania, 95131 Catania, Italy; abiondi@unict.it; 4 IRCCS Policlinico San Donato, Division of General and Foregut Surgery, Department of Biomedical Sciences for Health, University of Milan, 20097 Milan, Italy; luigi.bonavina@unimi.it

**Keywords:** gastric cancer, total gastrectomy, short-term outcomes, postoperative complications, functional outcomes

## Abstract

**Simple Summary:**

Minimally invasive approaches to total gastrectomy (TG) are gaining acceptance worldwide for the treatment of gastric cancer. Previous studies comparing short-term results were limited by pairwise comparisons and the inclusion of both total and distal gastrectomy. The present analysis aimed to compare the short-term outcomes of different techniques for TG comprehensively in the setting of GC. Lap-assisted TG (LATG), totally laparoscopic TG (TLTG), and robotic TG (RTG) seem to be associated with a lower overall complications rate and improved short-term functional outcomes compared to open TG (OTG). Severe postoperative complications and anastomotic leak seem comparable across techniques. Our findings support the adoption of minimally invasive TG techniques in the surgical management of gastric cancer.

**Abstract:**

Background/Objectives: Total gastrectomy (TG) is the cornerstone treatment for gastric cancer (GC). While open TG (OTG) with D2 lymphadenectomy remains the gold standard, alternative techniques such as lap-assisted TG (LATG), totally laparoscopic TG (TLTG), and robotic TG (RTG) have been reported with promising outcomes. The present analysis aimed to compare the short-term outcomes of different techniques for TG comprehensively in the setting of GC. Methods: A systematic review and network meta-analysis were performed. The primary outcomes were overall complications (OC), severe postoperative complications (SPCs), and anastomotic leak (AL). Pooled effect-size measures included risk ratio (RR), weighted mean difference (WMD), and 95% credible intervals (CrIs). Results: Sixty-eight studies (44,689 patients) were included. Overall, 52.4% underwent OTG, 6.5% LATG, 39.2% TLTG, and 1.9% RTG. Both TLTG (RR 0.82; 95% CrI 0.73–0.92) and RTG (RR 0.75; 95% CrI 0.59–0.95) showed a reduced rate of postoperative OC compared to OTG. SPCs and AL RR were comparable across all techniques. Despite the longer operative time, LATG, TLTG, and RTG showed reduced intraoperative blood loss, time to first flatus, ambulation, liquid diet resumption, and hospital stay compared to OTG. Conclusions: Minimally invasive approaches seem to be associated with improved OC and functional outcomes compared to OTG.

## 1. Introduction

Gastric cancer (GC) ranks fifth globally in incidence among malignancies and is the fifth leading cause of cancer-related mortality [1]. Surgery represents the cornerstone of curative-intent therapy for GC. Total gastrectomy (TG) is preferred over distal gastrectomy when tumor location precludes adequate proximal resection margins or when the invasion of an adjacent organ is reported [2]. Open total gastrectomy (OTG) with D2 lymphadenectomy is the gold standard treatment for locally advanced GC [3]. Since the description of the first laparoscopic and robotic TG, these minimally invasive approaches have gained increasing consensus worldwide, given improved functional outcomes and similar oncologic results [4,5,6,7,8,9,10]. 

Multiple studies and meta-analyses have previously been published to compare surgical techniques for TG. These articles were limited by the pairwise comparisons and by selection bias because of the inclusion of both TG and distal gastrectomy. Therefore, a comprehensive network analysis focusing on TG and simultaneously comparing all surgical approaches for TG is lacking [11,12].

The aim of the present network meta-analysis was to compare the short-term outcomes of different surgical approaches for TG in the setting of GC.

## 2. Materials and Methods

A systematic review was conducted following the preferred reporting items for systematic review and meta-analyses (PRISMA) guidelines [13]. The relevant literature was searched in scientific databases, including Scopus, PubMed, MEDLINE, Web of Science, Cochrane Central Library, Google Scholar, and ClinicalTrials.gov, accessed on 1 AUgust 2024 [14]. The search strategy employed both medical subheadings (MeSH) and truncated search words, with the Boolean operators AND/OR, employing the following terms: stomach cancer, total gastrectomy, laparoscopic total gastrectomy, laparoscopic assisted total gastrectomy, open total gastrectomy, robot assisted total gastrectomy, postoperative complication, severe postoperative complication, anastomosis leakage, short-term outcome, bleeding, anastomotic stenosis, duodenal stump leakage, pancreatic complications, pulmonary complications, surgical site infection, and thrombotic complication. The complete search strategy is reported in the Appendix A. Articles published up to 1 April 2024 were considered for inclusion, as well as the reference lists of the included studies. Ethical approval was not required for this study. This study was registered on PROSPERO (CRD42024558026).

### 2.1. Eligibility Criteria

Studies that provided data on short-term outcomes in patients who underwent TG for GC were examined for eligibility. We included all studies that compared OTG, lap-assisted TG (LATG), totally laparoscopic TG (TLTG), and RTG. Studies with potential overlapping populations were identified, and those with broader inclusion criteria were considered. Exclusion criteria comprised the following: (1) studies that did not report at least one of the predefined primary outcomes; (2) non-comparative analyses; (3) articles not written in English; (4) studies reporting mixed data, including other surgical procedures; and (5) articles with fewer than 5 patients per treatment arm.

### 2.2. Selection Process

The literature review was conducted independently by two authors (MM, DS) based on the predefined inclusion and exclusion criteria. Following the removal of duplicates, all articles underwent title screening. Those meeting the inclusion criteria underwent full-text review after abstract screening. Disagreements were resolved by two additional authors (AA, DB), blinded to the initial assessments.

### 2.3. Data Collection Process

Data collection was carried out by three independent authors (MM, AA, DS) who completed pro forma tables on Google Sheets containing predetermined variables. Collected variables include the following: author, publication year, country, study design, study period, patients demographics (number of patients, age, sex, body mass index (BMI), and American Society of Anesthesiologists physical status), tumor characteristics (location, histology, neoadjuvant and adjuvant therapy, and tumor size), surgical variables (surgical approach, anastomotic technique, lymphadenectomy extension, pathologic tumor staging, residual tumor classification, operative time, intraoperative blood loss, conversion, and number of lymph nodes retrieved), and postoperative short-term outcomes (anastomotic leak, overall complications, severe postoperative complications, in-hospital mortality, time to first flatus, time to first liquid intake, time to first ambulation, hospital length of stay, reintervention, transfusion requirement, bleeding, anastomotic stenosis, duodenal stump leak, pancreatic complications, pulmonary complications, thrombotic events, and surgical site infections). Following data collection, two other authors (DB, GB) compared all data at the conclusion of the review process to identify and resolve any discrepancies.

### 2.4. Outcomes of Interest and Definitions

Anastomotic leak (AL), overall postoperative complications (OC), and severe postoperative complications (SPCs) were the primary outcomes. AL was defined as radiographic evidence of contrast extravasation observed on a postoperative swallow study and/or computed tomography scans, endoscopic visualization of anastomotic dehiscence or fistula, or surgical drain output consistent with saliva. Postoperative complications were graded according to the Clavien–Dindo classification system, as follows: Grade 0, representing no complications; Grade 1, indicating any deviation from normal postoperative course without requiring medical intervention; Grade 2, involving complications necessitating pharmacological treatment; Grade 3, when complications required surgical, endoscopic, or radiological intervention (subcategorized as 3a for interventions not requiring general anesthesia and 3b for those requiring general anesthesia); Grade 4, in case of life-threatening complications necessitating intensive care unit management; and Grade 5, representing death [15]. SPCs were defined as those graded as Clavien–Dindo complications equal to or greater than 3. Secondary outcomes were operative time (OT, minutes), intraoperative blood loss (ml), number of retrieved lymph nodes, conversion to OTG, reintervention, postoperative bleeding requiring transfusion, anastomotic stenosis, duodenal stump leak, ileus, pancreatic complications, pulmonary complications, thrombotic events, surgical site infections, in-hospital mortality, time to first flatus (days), time to first liquid intake (days), time to ambulation (days), and hospital length of stay (LOS, days). TLTG was defined as a minimally invasive laparoscopic technique for gastric resection with intracorporeal anastomosis. LATG was outlined for laparoscopic gastric resection with extracorporeal anastomosis. RTG was characterized as robotic gastric resection, irrespective of the location of anastomosis formation.

### 2.5. Quality Assessment

Methodological quality assessment was conducted by three authors (MM, AA, GB) utilizing the appropriate evaluation tools. Observational studies underwent assessment using the ROBINS-I tool, which evaluates domains such as confounding bias, selection bias, classification bias, intervention bias, missing data bias, outcomes measurement bias, and reporting bias [16]. Each domain was rated as “Low”, “Moderate”, “Serious”, or “Critical”, and overall judgment categories for each study included low, moderate, serious, or critical risk of bias. Randomized controlled trials were evaluated using the version 2 of the Cochrane risk-of-bias tool for randomized trials, focusing on bias arising from the randomization process, bias due to deviations from intended interventions, bias due to missing outcome data, bias in measurement of the outcome, and bias in selection of the reported result [17]. Trials were subsequently classified as having overall low risk (green circle), high risk (red circle), or unclear risk (yellow circle) of bias.

### 2.6. Statistical Analysis

We performed an arm-based random effect frequentist network meta-analysis [18]. For dichotomous variables, the risk ratio (RR) was chosen as the effect size. For continuous variables, the standardized mean difference (SMD) was chosen as the effect size. A generalized DerSimonian–Larid [19] estimator was used to estimate the between-study variance, assumed as common for each pairwise treatment comparison. A generalized I^2^ was adopted to define heterogeneity as follows: low (<25%), moderate (25–75%), or high (>75%) [20]. To account for transitivity, the eligibility criteria of the included studies are framed in such a manner that the trials are primarily different in the tested interventions only. To assess transitivity, we generated descriptive statistics and compared the distributions of baseline characteristics across studies and treatment comparisons [21]. The node split was used as an assessment for the local inconsistency. The treatment ranking probability was estimated with the cumulative ranking curve (SUCRA). The network geometry was appraised, and the confidence of outcomes estimates was assessed with the Confidence in Network Meta-Analysis (CINeMA) instrument [22]. Two-sided *p*-values were considered statistically significant when less than 0.05, and the confidence intervals (CI) were computed at 95%. All analyses and graphs were carried out using R-CRAN statistical software (version 4.3.0) with the netmeta package [23].

## 3. Results

### 3.1. Systematic Review

**Figure 1:**
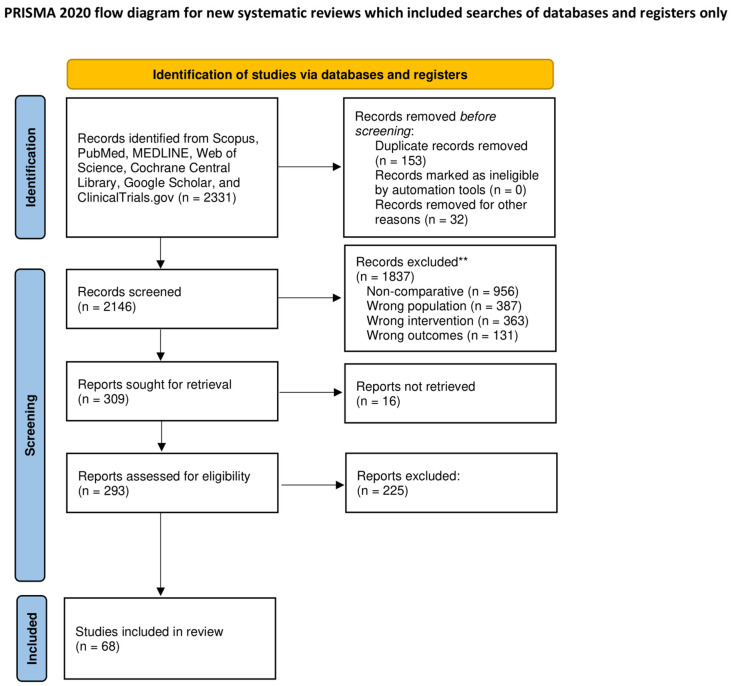
The preferred reporting items for systematic reviews checklist (PRISMA) diagram. **: lf automation tools were used, indicate how many records were excluded by a human and how many were excluded by automation tools.

The PRISMA flow diagram is depicted in Figure 1. The initial search strategy identified 2331 records. After duplicates removal, title and abstract screening, 293 articles were included for full-text evaluation. After full-text review, 68 articles were deemed eligible and included in the quantitative analysis. The study design was observational retrospective (60 studies, 25 of which employed propensity score matching), observational prospective (6 studies, 2 of which employed propensity score matching), and experimental (2 randomized controlled trials). The quality of the included studies is described in Table 1, Supplementary Figure S1, and Supplementary Table S1.

Baseline characteristics are shown in Table 1. Overall, data from 44,689 patients undergoing TG were analyzed. Surgical procedures were performed between 1995 and 2021. The surgical techniques were OTG (*n* = 23,414; 52.4%), LATG (*n* = 2918; 6.5%), TLTG (*n* = 17,499; 39.2%), and RTG (*n* = 858; 1.9%). The patient age ranged from 50.6 to 75 years, 73% were males, and preoperative BMI ranged between 19 and 32.1. Tumor location was specified in 39 studies and defined in the upper (68%), middle (29%), and distal (3%) portion of the stomach. Pathologic tumor staging was available in 51 studies, with pStage 0–I, II–III, and IV diagnosed in 27.8%, 71.9%, and 26% of cases, respectively. Neoadjuvant treatments were defined in 38 studies (9673 patients) and completed in 17.6% of subjects (1707 patients).

### 3.2. Meta-Analysis

#### 3.2.1. Primary Outcomes

**Figure 2:**
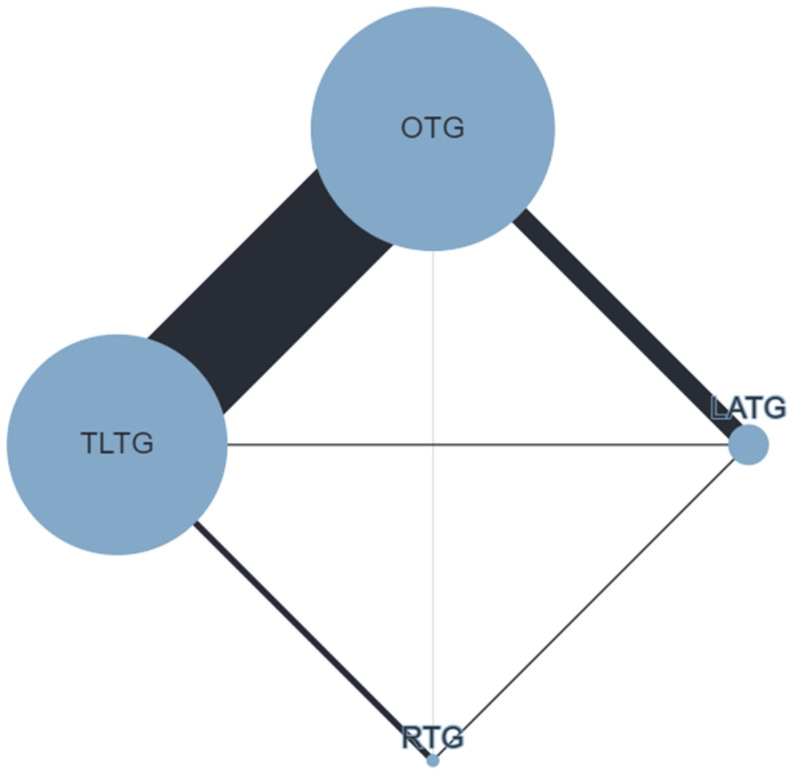
Network geometry for postoperative overall complications (OC). Node sizes reflect the sample size, while edge widths reflect the number of studies for a specific pairwise comparison.

**Figure 3:**
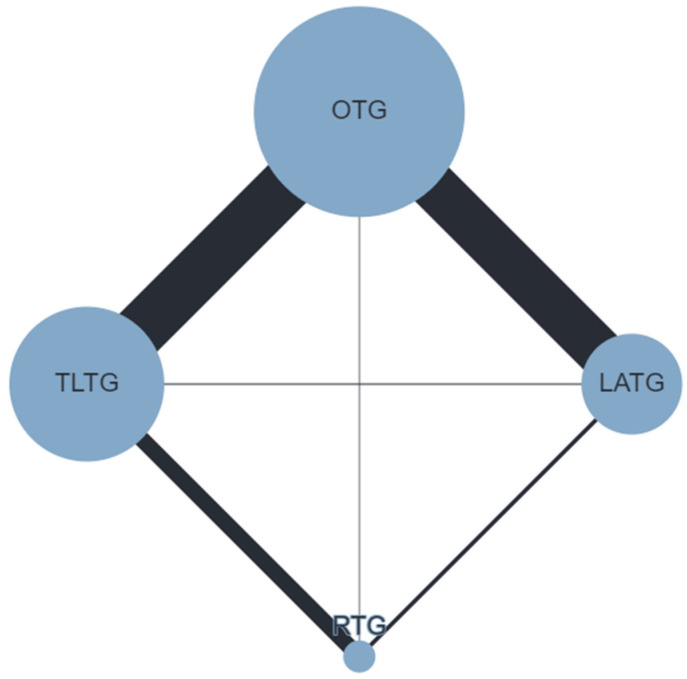
Network geometry for postoperative severe complications (SPCs). Node sizes reflect the sample size, while edge widths reflect the number of studies for a specific pairwise comparison.

**Figure 4:**
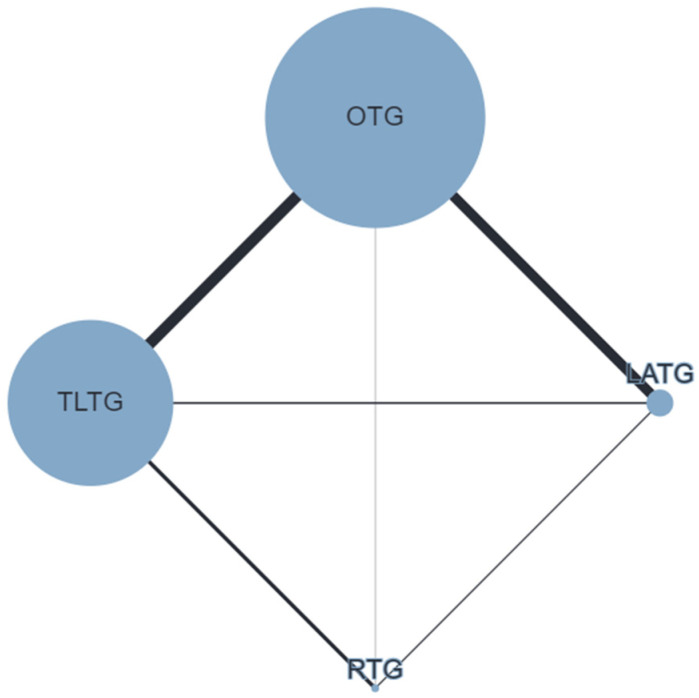
Network geometry for postoperative anastomotic leak (AL). Node sizes reflect the sample size, while edge widths reflect the number of studies for a specific pairwise comparison.

The postoperative OC rate (Figure 2) was defined in 65 studies (38,459 patients). TLTG (RR 0.82; 95% CrI 0.73–0.92) and RTG (RR 0.75; 95% CrI 0.59–0.95) were associated with reduced OC rates compared to OTG; no differences were observed for LATG vs. OTG (RR 0.92; 95% CrI 0.81–1.04) and TLTG vs. RTG (RR 1.09; 95% CrI 0.88–1.36). SPCs (Figure 3) were reported in 46 studies (10,306 patients). Compared to OTG, no differences were observed for LATG (RR 0.80; 95% CrI 0.59–1.07), TLTG (RR 0.96; 95% CrI 0.75–1.24), and RTG (RR 1.2; 95% CrI 0.75–1.91). AL (Figure 4) was conveyed in 61 studies (43,452 patients). No difference was observed for LATG vs. OTG (RR 1.15; 95% CrI 0.83–1.59), TLTG vs. OTG (RR 1.16; 95% CrI 0.95–1.43), and RTG vs. OTG (RR 1.27; 95% CrI 0.74–2.18). The treatment ranking evaluation showed that RTG had the lowest probability for SPCs (15%) and AL (31%).

#### 3.2.2. Secondary Outcomes

Conversion rate (23 studies, 2390 patients) was significantly lower for LATG vs. TLTG (RR 0.32; 95% CrI 0.11–0.92) and LATG vs. RTG (RR 0.40; 95% CrI 0.21–0.74). OT (65 studies, 14,365 patients) was significantly shorter for OTG compared to LATG (SMD −0.95; 95% CrI −1.41; −0.48), TLTG (SMD −1.14; 95% CrI −1.57; −0.71), and RTG (SMD −2.02; 95% CrI −2.74; −1.30); RTG showed longer OT vs. LATG (SMD 1.07; 95% CrI 0.31; 1.84) and TLTG (SMD 0.88; 95% CrI 0.23; 1.53). Intraoperative blood loss (56 studies, 11,983 patients) was significantly higher in OTG compared to LATG (SMD 1.15; 95% CrI 0.76; 1.54), TLTG (SMD 1.43; 95% CrI 1.08; 1.78), and RTG (SMD 1.68; 95% CrI 1.08; 2.28). LATG showed fewer lymph nodes retrieved compared to OTG (SMD −0.22; 95% CrI −0.39; −0.04), TLTG (SMD −0.28; 95% CrI −0.49; −0.06), and RTG (SMD −0.44; 95% CrI −0.74; −0.15). Postoperative bleeding requiring transfusion (48 studies, 16,598 patients) was significantly higher for TLTG vs. OTG (RR 1.28; 95% CrI 1.05–1.57). No differences were observed for anastomotic stenosis, duodenal stump leak, pancreatic complications, pulmonary complications, surgical site infections, postoperative thrombotic events, postoperative transfusion, postoperative ileus, reintervention, and in-hospital mortality among treatments.

#### 3.2.3. Functional Results

LOS (64 studies, 44,076 patients) and time to first flatus (36 studies, 8084 patients) were comparable between LATG, TLTG, and RTG; OTG showed longer HLOS and time to first flatus when compared to LATG (SMD 0.46; 95% CrI 0.21; 0.71; and SMD 0.97; 95% CrI 0.61; 1.33 respectively), TLTG (SMD 0.55; 95% CrI 0.33; 0.77; and SMD 0.71; 95% CrI 0.38; 1.04 respectively), and RTG (SMD 0.84; 95% CrI 0.45; 1.22; and SMD 1.22; 95% CrI 0.59; 1.85 respectively). Time to first liquid intake (28 studies, 31,465 patients) and time to first ambulation (14 studies, 3530 patients) were shorter for TLTG compared to OTG (SMD −0.87; 95% CrI −1.52; −0.21; and SMD −0.81; 95% CrI −1.52; −0.09, respectively). Descriptive statistics and the league table are depicted in Table 2 and Table 3, respectively. The node split analysis did not show evidence of inconsistence. The leverage plots did not show evidence of study outliers into this network meta-analysis. For all outcomes, there was no evidence of non-MCMC convergence using the diagnostic tools described in the statistical analysis section. The assessments of confidence in the estimates using CINeMA show moderate-to-very-low confidence, essentially due to study limitation, imprecision, and inconsistence.

## 4. Discussion

The present study shows comparable SPCs and AL RR for OTG, LATG, TLTG, and RTG. Despite longer OT, LATG, TLTG, and RTG showed reduced OC, intraoperative blood loss, time to first flatus, time to ambulation, time to liquid diet resumption, and HLOS compared to OTG.

GC incidence is progressively increasing in both low- and high-risk countries, with risk factors ranging from genetic susceptibility and Helicobacter pylori infection to lifestyle factors such as alcohol consumption and smoking [92]. In Western countries, GC is often diagnosed at an advanced stage due to inadequate screening protocols [93]. For individuals with resectable GC, a multimodal approach involving surgery alongside systemic therapy appears to confer survival benefits [94]. The backbone of curative treatment for GC is surgical resection [95]. The optimal surgical approach is still a matter of debate, with international guidelines stating that open total or distal gastrectomy are the current gold standard for clinically node-positive or T2–T4a tumors [2,3]. OTG has been flanked by minimally invasive approaches with promising results [24,96,97,98]. The technical advantages of these techniques with minimized surgical trauma have driven their wider worldwide adoption [10,29,99,100]. This shift from open surgery to minimally invasive approaches has been recently documented in a Korean nationwide survey; specifically, the frequency of the open approach decreased from 49.8% to 27.6% between 2014 and 2019. On the contrary, laparoscopic TG frequency increased by 18.2% in 2014 to 44.3% in 2019 (26.1%) [101,102].

Our network analysis showed OC rates of 18/% for OTG and LATG, 17% for TLTG, and 16% for RTG. These results are similar to the CLASS-02 trial, which reported OC rates of 19.1% and 20.2% for laparoscopic and open gastrectomy, respectively [48]. Interestingly, the quantitative analysis showed higher OC RR for OTG compared to TLTG and RTG with a low related heterogeneity (I^2^ = 22.5%). Our data are similar to a recent Korean analysis that observed a lower overall morbidity rate for minimally invasive vs. open gastrectomy (17.5% vs. 21.9%, *p* = 0.001) [103]. Similarly, Lei et al., in recently published RCTs meta-analysis, showed a lower OC rate for laparoscopic compared to open gastrectomy (OR 0.65, *p* < 0.001) [4]. These results may be theoretically explained by the reduced surgical trauma for minimally invasive gastrectomy with smaller surgical incisions, less surgical stress, and finest surgical dissection determining a lower risk of postoperative SSI and bleeding [104]. Interestingly, the postoperative SPCs RR were comparable across treatments. Our results are similar to those presented in the recent JLSSG0901 RCT, which reported no differences in SPCs between open (4.7%) and laparoscopic-assisted distal gastrectomy (4.7% vs. 3.5%; *p* = 0.641) [105]. Conversely, Wang et al. reported a lower SPCs rate for laparoscopic compared to robot-assisted gastrectomy (8.9% vs. 17.5%, *p* = 0.002) [106]. The incidence of AL after total gastrectomy for GC has been previously reported to be up to 6.6% [107]. The present analysis showed that OTG, LATG, TLTG, and RTG were associated with 8%, 6%, 4%, and 2% AL rates, respectively. Again, the quantitative analysis showed no significant RR differences among treatments. Our results are consistent with Yang et al., who reported comparable AL for laparoscopic TG vs. OTG (OR 0.94; 95% CI 0.61–1.47) [108]. Despite the related heterogeneity being low–moderate for these primary outcomes, selection bias, temporal bias, and reporting bias should be pondered while interpreting these outcomes. The surgical technique use to perform TG seems to have no influence on AL. Contrarily, AL may depend on other factors such as anastomotic tension, malnutrition, inadequate blood supply, and comorbidities [109].

In our meta-analysis, OTG exhibited the shortest OT. This is in contrast to what was reported by Garbarino et al. and Trastulli et al., which conveyed longer OT for open gastrectomy compared to laparoscopic (WMD 47.4 min; *p* < 0.001) and robotic gastrectomy (WMD 56.9 min; *p* < 0.001) [110,111]. These results mandate thoughtful insights as it is well known that in case of minimally invasive approaches, OT encompass both “effective” surgical time (dissection and reconstruction phases) and “junk time”, which involves setting, docking, and surgical instruments adjusting. Liu et al. previously reported comparable effective operative for robotic and laparoscopic distal gastrectomy (145.9 vs. 130.6; *p* = 0.09) [112]. Similarly, Omori et al. reported shorter OT for robotic compared to laparoscopic gastrectomy in a propensity-matched cohort of 1189 patients [113]. It has been shown that longer OT seems to be associated with an increased risk of postoperative complication [114]. Specifically, Park et al. identified 240 min as the cut-off associated with an increased risk of postoperative complications [115]. Interestingly our analysis reported 208, 248, 240, and 297 min estimated OT for OTG, LATG, TLTG, and RTG, respectively. These data suggest that even if a statistically significant difference is perceived, its clinical relevance may be limited. Further, OTG was associated with longer HLOS compared to minimally invasive approaches. These results are similar to Straatman et al., who reported shorter time to first flatus (WMD −1.05 days, *p* < 0.00001) and shorter HLOS (WMD: −2.43 days, *p* = 0.0002) for minimally invasive compared to open gastrectomy [116]. No significant differences were found in terms of harvested lymph nodes among techniques. These results reflect what was reported by Yang et al., which conveyed no significant difference between laparoscopic and open TG [108]. Conversely, Trastulli et al. stated a statistically significant higher number of lymph nodes harvested in robotic vs. open gastrectomy (30 vs. 25.5, *p* = 0.014) [111]. Additionally, a recent meta-analysis showed a higher number of lymph nodes retrieved in the robotic compared to laparoscopic gastrectomy (OR: 1.75; *p* < 0.0001) [117]. Minimally invasive approaches were associated with a trend toward improved time to first flatus, time to first liquid intake, and time to ambulation. These results may reflect the minimized abdominal wall and bowel surgical trauma determining reduced perceived pain with earlier mobilization, ambulation, and time to fist flatus. Notably, the global heterogeneity for secondary outcomes was moderate–high. Factors that could have influenced this issue include patients’ comorbid conditions, body mass index, ASA grade, smoking status, postoperative antibiotic therapy, outcome reporting, tumor types and size, technique for intestinal reconstruction, lymphadenectomy (D1 vs. D1+ vs. D2), omentectomy (total versus partial versus non-performed), hospitals protocols, implementation of enhanced recovery after surgery protocols, surgeons’ experience, and hospital volumes.

It has been postulated that the operating surgeon’s learning curve and expertise have a significant influence on short-term outcomes and could be a notable source of background bias. Specifically, operating-surgeon-related factors are critical in determining operative time, intraoperative blood loss, total number of harvested lymph nodes, and overall complications [118]. Chan et al. reported that the learning curve for minimally invasive approaches to TG is approximately 44 cases for TLTG and 21 cases for RTG. Similarly, Seika and colleagues conveyed that 44 cases are required for experienced laparoscopic surgeons to achieve LATG technical competency [119,120,121]. In the present meta-analysis, only a few studies specifically reported the operating surgeon’s proficiency; therefore, our conclusions should be cautiously interpreted.

To the best of our knowledge, this is the first network meta-analysis reporting outcomes exclusively for TG and performing a comprehensive analysis on all the principal techniques for TG. The previous literature on gastrectomy in GC is based mainly on pairwise analyses and studies evaluating results from both total and distal gastrectomy. On the plea for a granular analysis of short-term outcomes focused on TG, we performed an analysis of a homogeneous population of patients undergoing TG for GC. In the present analysis, network meta-analytic methods allowed us to combine data from a large number of studies with the assessment of both direct/indirect comparisons and treatment ranking. Some limitations should be assessed in interpreting our results. First, surgical data are derived from a long time span, from 1995 to 2021 (temporal bias), thus leading to possible differences in both the surgical environment and perioperative systemic therapy protocols with related nonhomogeneous postoperative complication risks. Second, non-uniform measuring methods for postoperative outcomes were seen among different studies, thus leading to possible detection bias. Third, the inclusion of both early and locally advanced GC can lead to potential selection bias. Because data were reported as aggregated, a robust stratified analysis was not feasible. Fourth, all procedures were performed by experienced surgeons in high-volume referral centers, representing the best predictable outcomes; applicability of these results should be considered in light of the surgeon’s proficiency and learning curve. Only a few studies specifically analyzed RTG outcomes; therefore, the available evidence remains preliminary and limited. Hence, no definitive and robust conclusions can currently be drawn regarding the safety and efficacy of this approach. Lastly, there was significant heterogeneity among the included studies in terms of postoperative management, rehabilitation protocols, and the application of enhanced recovery after surgery pathways.

## 5. Conclusions

LATG, TLTG, and RTG seem to be associated with improved OC, time to first flatus, time to first liquid intake, and time to ambulation compared to OTG in the setting of GC. Our network meta-analysis supports the implementation of minimally invasive TG techniques in the surgical treatment of GC.

## Figures and Tables

**Table 1:** Demographic and clinical characteristics of included patients.

Author, Year *Country*	Study Design	Period	Approach	No. pts	Age (yrs)	Sex (M)	BMI (kg/m^2^)	Location (P-M-D)	pStage 0–I	pStage II	pStage III	pStage IV	Neoadj/Periop tp	QoE
Eom, 2022 *Korea* [24]	Ret PSM	2012–2016	OTG	119	60.2 ± 11.6	94	23.8 ± 3.3	96-14-9	93	17	9	0	0	S
			TLTG	119	62.3 ± 11.4	93	23.3 ± 3.1	105-14-0	93	17	9	0	0	
Illuminati, 2023 *Italy* [25]	Ret	2010–2021	OTG	109	57.7 ± 8.5	83	20.8 ± 2.8	nr	0	8	92	0	36	S
			LATG	98	59.4 ± 8.5	69	21.1 ± 2.7		0	7	101	0	40	
Jia, 2023 *China* [26]	Ret PSM	2014–2021	RTG	147	62.9 ± 10	118	25 ± 3.7	90-57-0	44	41	62	0	0	M
			TLTG	371	62.5 ± 9.4	294	24.5 ± 3.4	239-132-0	121	92	158	0	0	
Kinoshita, 2022 *Japan* [27]	Ret PSM	2008–2018	OTG	163	67 ± 3.5	134	22.4 ± 1.1	nr	56	54	53	0	17	M
			TLTG	163	69 ± 3	130	22.2 ± 0.9		91	33	39	0	16	
Salvador-Roses, 2023 *Spain* [28]	Ret	2014–2021	OTG	48	64 ± 13	40	27 ± 4	nr	nr	nr	nr	nr	26	S
			RTG	30	68 ± 13	23	26 ± 5						13	
Zheng, 2023 *China* [29]	Ret	2008–2018	OTG	57	63.3 ± 11.3	48	nr	nr	6	15	36	0	57	M
			TLTG	89	60.3 ± 10.1	69			16	35	38	0	89	
Hu, 2022 *China* [30]	Ret PSM	2011–2018	OTG	69	53.9 ± 12.7	52	22.8 ± 3.3	22-37-10	0	22	44	3	69	S
			TLTG	69	53.4 ± 13.4	53	22.6 ± 3.1	28-34-7	0	27	38	4	69	
Hikage, 2022 *Japan* [31]	Ret	2013–2020	RTG	36	72 ± 8	26	23.1 ± 2	nr	25	5	6	0	nr	S
			TLTG	58	71 ± 9	46	22.8 ± 3.2		42	15	1	0		
Chen, 2022 *China* [32]	Pro PSM	2015–2020	RTG	48	61.3 ± 9.3	38	22.3 ± 2.7	31-17-0	13	16	19	0	nr	L
			TLTG	96	61.6 ± 7.6	79	22.3 ± 3.2	66-30-0	22	29	45	0		
Cui, 2022 *China* [33]	Ret	2012–2019	OTG	75	56.8 ± 11.9	59	23.7 ± 3.3	46-29-0	24	16	34	1	75	S
			TLTG	61	57.6 ± 10.4	47	22.8 ± 2.7	40-21-0	9	22	29	1	61	
Di Carlo, 2022 *Italy* [34]	Ret	2015–2021	OTG	53	71 ± 5.8	33	24.5 ± 2.9	nr	0	23	30	0	44	S
			TLTG	39	67 ± 6.5	25	22.6 ± 2.2		0	18	21	0	30	
Li, 2022 *China* [35]	Pro	2018–2021	RTG	69	59.4 ± 9.9	48	22.6 ± 2.8	50-13-6	14	17	38	0	nr	L
			TLTG	73	58.9 ± 9.2	52	22.9 ± 2.8	52-16-5	15	19	39	0		
Lin, 2023 *China* [36]	Ret PSM	2014–2018	LATG	208	nr	140	nr	68-106-34	59	64	85	0	208	S
			TLTG	104		71		32-57-15	29	30	45	0	104	
Qiu, 2022 *China* [37]	Ret	2020	LATG	51	63.9 ± 8.2	36	23.1 ± 4.2	34-17-0	11	17	23	0	nr	M
			TLTG	46	63.3 ± 9.1	31	23.7 ± 4	31-15-0	11	15	20	0		
Shibasaki, 2022 *Japan* [38]	Ret PSM	2009–2021	RTG	100	69 ± 3.8	69	23 ± 1.3	nr	46	23	31	0	13	M
			TLTG	100	68 ± 3.5	67	23.1 ± 1.1		34	26	40	0	10	
Wang, 2022 *China* [39]	Ret PSM	2016–2020	RTG	115	60.4 ± 9.4	91	22.5 ± 3	85-30-0	30	40	45	0	108	L
			TLTG	230	60.3 ± 10.4	179	22.4 ± 3	172-58-0	72	60	98	0	214	
van der Wielen, 2021 *Europe* [40]	RCT	2015–2018	OTG	49	61.8 ± 10	32	25.2 ± 4	14-25-10	nr	nr	nr	nr	49	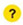
			TLTG	47	59.4 ± 12.5	28	26.5 ± 4.8	13-25-9					47	
Challine, 2021 *France* [41]	Ret	2013–2018	OTG	5037	68 ± 4.5	nr	nr	nr	nr	nr	nr	nr	nr	S
			TLTG	745	64 ± 5									
Fan, 2021 *China* [42]	Ret PSM	2011–2018	OTG	131	nr	97	nr	90-41-0	23	40	68	0	12	M
			LATG	131		98		93-38-0	24	41	66	0	14	
Feng, 2021 *China* [43]	Ret PSM	2011–2015	OTG	225	61 ± 3.5	164	22.2 ± 1	nr	18	42	165	0	nr	M
			TLTG	225	59 ± 4.3	154	21.9 ± 1		8	71	146	0		
Ko, 2021 *Korea* [44]	Ret PSM	2012–2018	OTG	61	58.7 ± 10.7	41	24 ± 2.7	nr	33	19	9	0	0	S
			TLTG	61	58.3 ± 11.3	40	24 ± 3		31	19	11	0	0	
Kumamoto, 2022 *Japan* [45]	Ret	2017–2021	RTG	27	69 ± 3.5	19	23.2 ± 0.7	2-25-0	nr	nr	nr	nr	2	S
			TLTG	29	70 ± 1.8	19	22.4 ± 0.9	7-22-0					7	
Roh, 2021 *Korea* [46]	Ret PSM	2009–2018	RTG	74	53.8 ± 11.6	42	23.6 ± 2.9	51-23-0	50	15	9	0	nr	M
			TLTG	74	54.6 ± 12.7	42	23.8 ± 3.4	47-27-0	47	18	9	0		
Wang, 2021 *China* [47]	Ret PSM	2013–2018	OTG	46	59.7 ± 8.7	36	23.3 ± 3.7	46-0-0	15	17	13	0	46	M
			TLTG	23	60.1 ± 9.7	18	23.7 ± 3.8	23-0-0	4	11	8	0	23	
Liu F, 2020 *China* [48]	RCT	2017–2018	OTG	109	59.4 ± 9.2	80	23.7 ± 3.1	nr	91	14	4	0	nr	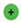
			TLTG	105	59.8 ± 9.4	75	23.9 ± 3.1		85	13	7	0		
Komatsu, 2020 *Japan* [49]	Ret PSM	200–2015	OTG	65	67	51	22	39-26-0	nr	nr	nr	nr	0	S
			TLTG	65	68	50	22	39-26-0					0	
Yang, 2020 *China* [50]	Ret PSM	2010–2017	RTG	126	60.3 ± 8.9	105	22.1 ± 2.5	58-68-0	3	30	93	0	0	M
			LATG	126	60.8 ± 9.1	100	22.1 ± 2.8	61-65-0	3	27	86	0	0	
Lee, 2020 *Korea* [51]	Ret PSM	2004–2014	OTG	51	63 ± 13.5	36	nr	26-25-0	1	23	27	0	0	M
			TLTG	51	62.1 ± 12.3	37		24-27-0	1	21	29	0	0	
Sakamamoto, 2020 *Japan* [52]	Ret PSM	2010–2017	OTG	12,229	nr	8998	nr	nr	nr	nr	nr	nr	nr	S
			TLTG	12,229		9005								
Zhao, 2019 *China* [53]	Ret PSM	2012–2017	OTG	217	59 ± 10.6	175	22.7 ± 2.6	217-0-0	2	122	93	0	9	M
			TLTG	468	60.4 ± 10.4	330	22.5 ± 2.6	468-0-0	7	268	193	0	61	
Ye, 2019 *China* [54]	Ret	2015–2018	RTG	99	58.7 ± 6.7	58	23.9 ± 1.9	33-66-0	2	54	43	0	0	M
			LATG	106	59 ± 7.3	55	23.9 ± 1.4	42-64-0	3	51	52	0	0	
Aoyama, 2018 *Japan* [55]	Ret	2011–2016	OTG	208	70 ± 9.2	154	nr	nr	nr	nr	nr	nr	nr	S
			LATG	95	69 ± 6.5	68								
Li, 2019 *China* [56]	Ret PSM	2008–2014	OTG	296	nr	200	22.5 ± 2.9	nr	47	119	130	0	0	L
			LATG	296		214	22.8 ± 3.2		47	119	130	0	0	
Wang, 2019 *China* [57]	Ret	2009–2014	OTG	43	61.9 ± 6	23	nr	43-0-0	17	21	5	0	0	S
			LATG	32	61.9 ± 8.7	21		32-0-0	11	17	4	0	0	
Etoh, 2018 *Japan* [58]	Pro PSM	2014–2015	OTG	512	nr	378	nr	nr	nr	nr	nr	nr	41	S
			TLTG	512		383							38	
Chen K, 2017 *China* [59]	Ret	2007–2016	OTG	124	53.5 ± 14.6	81	23 ± 3.7	nr	59	28	37	0	nr	L
			TLTG	124	52.7 ± 13.1	81	23.9 ± 4.3		60	29	35	0		
Chen XZ, 2017 *China* [60]	Ret PSM	2006–2015	OTG	69	60.5 ± 9.3	58	23 ± 3	37-19-13	14	16	38	1	nr	M
			LATG	69	57.1 ± 10.1	58	21.1 ± 2.1	41-21-7	14	16	38	1		
Lin JX, 2017 *China* [61]	Ret PSM	nr	OTG	346	61.3 ± 10.1	274	22 ± 2.3	188-136-22	51	62	233	0	nr	M
			LATG	346	61.1 ± 10	274	22 ± 2.9	191-136-19	46	53	247	0		
Kim EY, 2016 *Korea* [62]	Ret	2009–2014	LATG	29	59.3 ± 13.1	20	23.3 ± 3.2	17-12-0	12	6	10	1	nr	M
			TLTG	27	60.8 ± 9.1	22	24 ± 2.9	21-6-0	25	1	1	0		
Kim HB, 2016 *Korea* [63]	Ret	2013–2015	TLTG	30	51 ± 12.3	16	22.2 ± 2.7	19-11-0	nr	nr	nr	nr	nr	S
			LATG	24	53 ± 11.3	14	22.7 ± 11.8	12-12-0						
Wu H, 2016 *China* [64]	Ret PSM	2008–2013	OTG	74	60 ± 7.5	50	21 ± 1.8	nr	5	55	14	0	nr	M
			TLTG	74	62 ± 9.5	53	19 ± 1.5		6	53	15	0		
Shu B, 2016 *China* [65]	Ret	2007–2014	OTG	136	64 ± 5.2	92	21 ± 1.8	92-44-0	20	76	43	0	nr	M
			LATG	136	65 ± 5	86	20 ± 1.7	87-49-0	21	67	48	0		
Lu Y, 2016 *China* [66]	Ret	2008–2015	OTG	61	57 ± 6.8	37	22 ± 2	nr	8	36	17	0	0	M
			TLTG	61	59 ± 7.8	39	19 ± 1.3		6	39	16	0	0	
Huang, 2017 *China* [67]	Ret PSM	2007–2014	OTG	171	61.4 ± 10	152	21.9 ± 3	171-0-0	29	42	100	0	0	M
			LATG	171	62.4 ± 8.9	152	22.2 ± 2.9	171-0-0	27	47	97	0	0	
Park, 2016 *China* [68]	Pro	2011–2012	TLTG	30	57.1 ± 11.1	18	nr	nr	26	4		0	nr	S
			RTG	42	51.7 ± 12	26			28	14		0		
Shida, 2016 *Japan* [69]	Ret	2005–2013	OTG	53	65.5 ± 12.3	47	23.2 ± 3.6	40-13-0	32	15	6	0	0	M
			LATG	100	63.8 ± 11.3	84	23.6 ± 3.1	74-26-0	79	17	4	0	0	
Zhang, 2017 *China* [70]	Ret	2009–2012	OTG	85	72.9 ± 10.9	50	22.3 ± 2.5	73-12-0	nr	nr	nr	nr	nr	M
			LATG	69	69.4 ± 10.5	38	20.9 ± 2.1	57-12-0						
Ramagem CAG, 2015 *Brazil* [71]	Ret	2009–2013	OTG	64	60 ± 11.7	43	32.1 ± 4.1	6-27-31	21	16	27	0	0	M
			TLTG	47	58 ± 10.5	34	22.3 ± 4.4	4-24-19	14	13	20	0	0	
Lee, 2015 *Korea* [72]	Ret	2003–2010	OTG	502	57.6 ± 11.6	319	23.1 ± 11.6	371-131-0	nr	nr	nr	nr	0	S
			LATG	251	58.4 ± 12.7	160	23.1 ± 3	200-51-0					0	
Lu, 2015 *China* [73]	Ret PSM	2002–2012	OTG	252	nr	213	nr	183-69-0	56	45	151	0	0	M
			TLTG	252		208		177-75-0	52	56	144	0	0	
Shen, 2016 *China* [74]	Ret	2011–2014	TLTG	75	58.6 ± 11.6	57	24.4 ± 3.7	nr	23	21	31	0	nr	S
			RTG	23	57.3 ± 10.5	18	24.6 ± 3.5		10	4	9	0		
Song, 2015 *Korea* [75]	Ret	2009–2013	OTG	134	58.5 ± 12.3	94	22.7 ± 3.7	nr	nr	nr	nr	nr	nr	M
			TLTG	74	55.9 ± 11.7	45	22.9 ± 3							
Lee, 2014 *Korea* [76]	Ret	2006–2009	OTG	50	59 ± 4.1	39	nr	30-20-0	25	13	12	0	35	S
			LATG	34	61 ± 3.5	25		15-19-0	22	7	5	0	23	
Lee M, 2013 Korea [77]	Ret	2003–2010	OTG	50	51 ± 22.6	32	23 ± 3.4	nr	24	13	9	4	nr	S
			LATG	50	50.6 ± 22.1	32	23.2 ± 3.7		24	13	9	4		
Bo T, 2013 *China* [78]	Ret PSM	2004–2010	OTG	117	52.6 ± 13.6	80	21.7 ± 3.8	65-52-0	4	38	75	0	0	L
			LATG	117	54.5 ± 10.6	82	21.1 ± 3	64-53-0	6	40	71	0	0	
Guan G, 2013 *China* [79]	Ret	2007–2010	OTG	56	57.8 ± 9.9	40	nr	nr	25	25	6	0	nr	M
			LATG	41	60.7 ± 9.1	33			18	20	3	0		
Kim HS, 2013 *Korea* [80]	Ret	2011	OTG	207	56 ± 8.8	134	24.1 ± 3.1	nr	nr	nr	nr	nr	nr	M
			TLTG	139	58 ± 9	86	23.6 ± 3.1							
Kim KH, 2014 *Korea* [81]	Ret PSM	2002–2010	OTG	60	56.7 ± 12.4	36	22.8 ± 3.3	nr	40	13	7	0	nr	M
			LATG	60	57.3 ± 13.2	35	22.6 ± 3.1		39	14	7	0		
Jeong O, 2013 *Korea* [82]	Ret PSM	2003–2011	OTG	122	62.6 ± 11.7	93	23.5 ± 3.2	87-35-0	99	16	7	0	0	S
			TLTG	122	63.2 ± 11.2	89	23.1 ± 3.4	94-28-0	105	13	4	0	0	
Hong, 2013 *China* [83]	Ret	2008–2012	OTG	104	54.5 ± 10.4	76	24.4 ± 1.2	104-0-0	5	54	45	0	0	S
			TLTG	100	53.2 ± 11.1	71	24.1 ± 2.3	100-0-0	6	53	41	0	0	
Eom BW, 2012 *Korea* [84]	Ret	2003–2008	OTG	348	58.7 ± 11.5	254	23.8 ± 2.9	nr	nr	nr	nr	nr	0	S
			LATG	100	54.9 ± 13.5	57	22.7 ± 2.8						0	
Kim M, 2011 *Korea* [85]	Ret	2009–2010	OTG	127	57.3 ± 11.1	81	23.0 ± 2.9	nr	nr	nr	nr	nr	nr	M
			LATG	63	55.9 ± 12.2	43	22.7 ± 2.5							
Yoon, 2012 *Korea* [86]	Ret	2009–2011	RTG	36	53.9 ± 11.7	18	23.2 ± 2.5	nr	29	7	0	0	0	M
			LATG	65	56.9 ± 12.3	31	23.6 ± 3.4		55	7	3	0	0	
Sakuramoto S, 2009 *Japan* [87]	Ret	2003–2007	OTG	44	67.2 ± 9.9	10	22.5 ± 3.6	28-16-0	15	17	12	0	nr	M
			LATG	30	63.7 ± 9.2	12	21.9 ± 2.7	18-12-0	25	2	3	0		
Kawamura, 2009 *Japan* [88]	Ret	2003–2008	OTG	35	65.2 ± 10.7	25	22.9 ± 2.4	35-0-0	35	nr	nr	nr	0	M
			TLTG	46	64 ± 10.4	36	22.8 ± 3	46-0-0	46	nr	nr	nr	0	
Mochiki, 2008 *Japan* [89]	Ret	1998–2007	OTG	18	63 ± 2.2	16	nr	15-3-0	nr	nr	nr	nr	nr	S
			LATG	20	66 ± 2.4	16		18-2-0						
Topal B, 2008 *Belgium* [90]	Pro + Ret	2003–2006	OTG	22	69 ± 12	17	nr	4-13-5	7	7	6	2	nr	S
			TLTG	38	68 ± 12	23		11-17-10	17	7	10	4		
Dulucq, 2005 *France* [91]	Pro	1995–2004	OTG	11	67 ± 14	5	nr	nr	nr	nr	nr	nr	nr	S
			TLTG	8	75 ± 8	3								

Study design retrospective (Ret); prospective (Pro); propensity score matched (PSM); randomized controlled trial (RCT); surgical approach (Approach); open total gastrectomy (OTG); lap-assisted total gastrectomy (LATG); totally laparoscopic total gastrectomy (TLTG); robotic total gastrectomy (RTG); number of patients (No. pts); male (M); body mass index (BMI); tumor location (Location); proximal–middle–distal (P-M-D); pathologic tumor staging according to 7th–8th American Joint Committee on Cancer or 13th–14th–15th Japanese Classification of Gastric Carcinoma (pStage); neoadjuvant therapy (Neoadj tp); perioperative therapy (Periop tp); quality of the evidence (QoE); according to ROBINS-I tool, Low-Moderate–Serious risk of bias (L-M-S); Cochrane risk-of-bias tool 2, low–unclear risk of bias (Green–Yellow circle); *NR* not reported. Data are reported as numbers, mean ± standard deviation.

**Table 2:** Descriptive statistics stratified according to different treatments.

	OTG	LATG	TLTG	RTG
OC	18 (5–46)	18 (5–41)	17 (0–39)	16 (3–25)
SPCs	7 (0–23)	6 (0–14)	6 (0–27)	6 (0–58)
AL	8 (0–27)	3 (0–10)	4 (0–28)	2 (0–25)
Anastomotic stenosis	2 (0–9)	3 (0–9)	1 (0–10)	4 (3–17)
Duodenal stump leak	1 (0–4)	1 (0–3)	1 (0–7)	2 (0–8)
Pancreatic complications	2 (0–6)	1 (0–6)	1 (0–5)	1 (0–3)
Pulmonary complications	8 (0–79)	5 (0–15)	5 (0–25)	7 (0–17)
SSI	4 (0–18)	3 (0–29)	2 (0–10)	2 (1–17)
Thrombotic events	2 (0–6)	0 (0–1)	1 (0–4)	2 (1–11)
Bleeding	5 (0–8)	2 (0–6)	4 (0–10)	2 (0–17)
Transfusion requirement	17 (6–27)	4 (0–7)	10 (0–19)	10 (4–16)
Ileus	2 (0–7)	1 (0–7)	2 (0–8)	2 (0–8)
Reintervention	15 (0–18)	1 (0–5)	9 (0–16)	2 (0–11)
In-hospital mortality	1 (0–9)	0 (0–2)	1 (0–3)	0 (0–8)
OT	208.9 (109.4–323)	240.5 (150.8–338.7)	248.3 (144–466)	297.5 (203.9–550)
Intraoperative blood loss	247.9 (99.2–758)	133.8 (50–299)	107.2 (10–275.3)	89 (32.5–207.1)
No LN retrieved	36 (15–54.3)	35 (18–48.4)	36.3 (18–54)	39.1 (22–48)
Time to first flatus	3.9 (3–5.4)	4.8 (2.4–23.4)	3.3 (1.9–5.7)	6.9 (2.2–23.1)
Time to first liquid intake	4.9 (2–10.7)	4.9 (3.5–9.1)	3.9 (1.5–9.1)	3.8 (3.5–3.9)
Time to first ambulation	3.7 (0.7–10.4)	3.6 (0.6–9.1)	2.2 (1.7–3.1)	2.3 (1.8–2.8)
LOS	14.2 (6–29)	10.8 (7–19)	13.1 (5–18)	9.8 (7–13)

Open total gastrectomy (OTG); lap-assisted total gastrectomy (LATG); totally laparoscopic total gastrectomy (TLTG); robotic total gastrectomy (RTG); overall complications (OC); severe postoperative complications (SPCs); anastomotic leak (AL); surgical site infections (SSI); operating time (OT); hospital length of stay (LOS). Values are presented as percentages for categorical variables and as mean (range) for continuous variables.

**Table 3:** League table. Each row represents a specific outcome.

Outcomes					I^2^ (95% CrI)
OC	**OTG**	0.92 (0.81–1.04)	0.82 (0.73–0.92)	0.75 (0.59–0.95)	22.5 (0–43.9)
	1.08 (0.96–1.23)	**LATG**	0.89 (0.76–1.04)	0.81 (0.63–1.05)	
	1.22 (1.09–1.36)	1.13 (0.96–1.32)	**TLTG**	0.92 (0.74–1.14)	
	1.33 (1.05–1.68)	1.23 (0.95–1.58)	1.09 (0.88–1.36)	**RTG**	
SPCs	**OTG**	0.80 (0.59–1.07)	0.96 (0.75–1.24)	1.20 (0.75–1.91)	27.5 (0–50.3)
	1.25 (0.93–1.69)	**LATG**	1.21 (0.84–1.75)	1.50 (0.91–2.49)	
	1.04 (0.81–1.34)	0.83 (0.57–1.20)	**TLTG**	1.24 (0.80–1.94)	
	0.84 (0.52–1.33)	0.67 (0.40–1.10)	0.80 (0.51–1.26)	**RTG**	
AL	**OTG**	1.15 (0.83–1.59)	1.16 (0.95–1.43)	1.27 (0.74–2.18)	17.6 (0–40.9)
	0.87 (0.63–1.20)	**LATG**	1.01 (0.70–1.47)	1.11 (0.61–2.01)	
	0.86 (0.70–1.05)	0.99 (0.68–1.43)	**TLTG**	1.09 (0.65–1.85)	
	0.79 (0.46–1.35)	0.90 (0.50–1.64)	0.92 (0.54–1.55)	**RTG**	
Anastomotic stenosis	**OTG**	1.46 (0.95–2.24)	1.25 (0.66–2.38)	2.95 (0.81–10.81)	0 (0–42.5)
	0.69 (0.45–1.05)	**LATG**	0.86 (0.42–1.77)	2.02 (0.57–7.21)	
	0.80 (0.42–1.51)	1.16 (0.57–2.39)	**TLTG**	2.35 (0.63–8.78)	
	0.34 (0.09–1.24)	0.49 (0.14–1.77)	0.43 (0.11–1.59)	**RTG**	
Duodenal stump leak	**OTG**	0.79 (0.40–1.57)	1.96 (0.64–5.98)	1.40 (0.42–4.66)	0 (0–52.3)
	1.26 (0.64–2.49)	**LATG**	2.47 (0.71–8.61)	1.77 (0.54–5.78)	
	0.51 (0.17–1.56)	0.40 (0.12–1.41)	**TLTG**	0.72 (0.16–3.18)	
	0.71 (0.21–2.37)	0.57 (0.17–1.85)	1.40 (0.31–6.22)	**RTG**	
Pancreatic complications	**OTG**	0.79 (0.40–1.57)	1.96 (0.64–5.98)	1.40 (0.42–4.66)	0 (0–40.2)
	1.26 (0.64–2.49)	**LATG**	2.47 (0.71–8.61)	1.77 (0.54–5.78)	
	0.51 (0.17–1.56)	0.40 (0.12–1.41)	**TLTG**	0.72 (0.16–3.18)	
	0.71 (0.21–2.37)	0.57 (0.17–1.85)	1.40 (0.21–6.22)	**RTG**	
Pulmonary complications	**OTG**	0.96 (0.74–1.24)	0.99 (0.91–1.08)	0.84 (0.56–1.26)	0 (0–33.8)
	1.04 (0.80–1.35)	**LATG**	1.03 (0.79–1.35)	0.88 (0.55–1.40)	
	1.01 (0.93–1.10)	0.97 (0.74–1.27)	**TLTG**	0.85 (0.57–1.26)	
	1.19 (0.79–1.78)	1.14 (0.71–1.83)	1.18 (0.79–1.75)	**RTG**	
SSI	**OTG**	0.58 (0.42–0.81)	0.90 (0.80–1.00)	1.13 (0.58–2.21)	0 (0–33.8)
	1.72 (1.24–2.40)	**LATG**	1.54 (1.09–2.18)	1.94 (0.98–3.85)	
	1.12 (1.00–1.25)	0.65 (0.46–0.92)	**TLTG**	1.26 (0.65–2.46)	
	0.89 (0.45–1.73)	0.51 (0.26–1.02)	0.79 (0.41–1.55)	**RTG**	
Thrombotic events	**OTG**	1.11 (0.25–4.99)	0.93 (0.70–1.24)	1.96 (0.59–6.56)	0 (0–42.5)
	0.90 (0.20–4.02)	**LATG**	0.83 (0.18–3.76)	1.76 (0.31–10.12)	
	1.08 (0.81–1.44)	1.2 (0.27–5.41)	**TLTG**	2.11 (0.65–6.86)	
	0.51 (0.15–1.70)	0.57 (0.10–3.26)	0.47 (0.15–1.54)	**RTG**	
Bleeding	**OTG**	1.05 (0.68–1.62)	1.28 (1.05–1.57)	1.72 (0.84–3.51)	0 (0–34.2)
	0.95 (0.62–1.47)	**LATG**	1.22 (0.77–1.95)	1.64 (0.75–3.57)	
	0.78 (0.64–0.95)	0.82 (0.51–1.30)	**TLTG**	1.34 (0.67–2.69)	
	0.58 (0.28–1.19)	0.61 (0.28–1.33)	0.75 (0.37–1.50)	**RTG**	
Transfusion requirement	**OTG**	0.40 (0.18–0.88)	0.75 (0.50–1.11)	0.78 (0.36–1.68)	57 (13–78.8)
	2.50 (1.13–5.51)	**LATG**	1.86 (0.77–4.52)	1.94 (0.64–5.86)	
	1.34 (0.90–1.99)	0.54 (0.22–1.30)	**TLTG**	1.04 (0.54–2.02)	
	1.29 (0.59–2.78)	0.52 (0.17–1.56)	0.96 (0.50–1.86)	**RTG**	
Ileus	**OTG**	0.88 (0.59–1.31)	0.97 (0.83–1.13)	1.03 (0.53–1.97)	0 (0–33.8)
	1.14 (0.76–1.69)	**LATG**	1.10 (0.73–1.67)	1.17 (0.56–2.41)	
	1.03 (0.89–1.20)	0.91 (0.60–1.37)	**TLTG**	1.06 (0.56–2.01)	
	0.98 (0.51–1.88)	0.86 (0.42–1.77)	0.95 (0.50–1.80)	**RTG**	
Conversion	**LATG**	3.10 (1.09–8.84)	2.52 (1.35–4.70)		0 (0–64.8)
	0.32 (0.11–0.92)	**TLTG**	0.81 (0.33–1.97)		
	0.40 (0.21–0.74)	1.23 (0.51–2.99)	**RTG**		
Reintervention	**OTG**	1.06 (0.27–4.16)	1.09 (0.67–1.77)	1.03 (0.38–2.77)	74.7 (61.7–83.3)
	0.94 (0.24–3.69)	**LATG**	1.02 (0.24–4.28)	0.97 (0.19–4.91)	
	0.92 (0.57–1.50)	0.98 (0.23–4.10)	**TLTG**	0.95 (0.40–2.27)	
	0.97 (0.36–2.60)	1.03 (0.20–5.19)	1.05 (0.44–2.51)	**RTG**	
In-hospital mortality	**OTG**	0.89 (0.46–1.74)	1.04 (0.80–1.36)	1.88 (0.70–5.11)	74.7 (61.7–83.3)
	1.12 (0.58–2.17)	**LATG**	1.17 (0.58–2.35)	2.11 (0.71–6.28)	
	0.96 (0.74–1.25)	0.86 (0.43–1.72)	**TLTG**	1.80 (0.67–4.89)	
	0.53 (0.20–1.44)	0.47 (0.16–1.42)	0.55 (0.20–1.50)	**RTG**	
OT	**OTG**	0.95 (0.48; 1.41)	1.14 (0.71; 1.57)	2.02 (1.30; 2.74)	98.3 (98.1–98.5)
	−0.95 (−1.41; −0.48)	**LATG**	0.19 (−0.37; 0.76)	1.07 (0.31; 1.84)	
	−1.14 (−1.57; −0.71)	−0.19 (−0.76; 0.37)	**TLTG**	0.88 (0.23; 1.53)	
	−2.02 (−2.74; −1.3)	−1.07 (−1.84; −0.31)	−0.88 (−1.53; −0.23)	**RTG**	
Intraoperative blood loss	**OTG**	−1.15 (−1.54; −0.76)	−1.43 (−1.78; −1.08)	−1.68 (−2.28; −1.08)	98.45 (87.3–99.2)
	1.15 (0.76; 1.54)	**LATG**	−0.27 (−0.73; 0.19)	−0.53 (−1.17; 0.12)	
	1.43 (1.08; 1.78)	0.27 (−0.19; 0.73)	**TLTG**	−0.25 (−0.78; 0.27)	
	1.68 (1.08; 2.28)	0.53 (−0.12; 1.17)	0.25 (−0.27; 0.78)	**RTG**	
No LN retrieved	**OTG**	−0.22 (−0.39; −0.04)	0.06 (−0.11; 0.22)	0.23 (−0.05; 0.51)	98.3 (77.3–98.4)
	0.22 (0.04–0.39)	**LATG**	0.28 (0.06; 0.49)	0.44 (0.15; 0.74)	
	−0.06 (−0.22; 0.11)	−0.28 (−0.49; −0.06)	**TLTG**	0.17 (−0.08; 0.42)	
	−0.23 (−0.51; 0.05)	−0.44 (−0.74; −0.15)	−0.17 (−0.42; 0.08)	**RTG**	
Time to first flatus	**OTG**	−0.97 (−1.33; −0.61)	−0.71 (−1.04; −0.38)	−1.22 (−1.85; −0.59)	90.4 (67.3–99.4)
	0.97 (0.61; 1.33)	**LATG**	0.26 (−0.16; 0.69)	−0.25 (−0.89; 0.38)	
	0.71 (0.38; 1.04)	−0.26 (−0.69; 0.16)	**TLTG**	−0.51 (−1.10; 0.07)	
	1.22 (0.59; 1.85)	0.25 (−0.38; 0.89)	0.51 (−0.07; 1.10)	**RTG**	
Time to first liquid intake	**OTG**	−0.46 (−1.35; 0.44)	−0.87 (−1.52; −0.21)	−1.20 (−2.54; 0.14)	99.3 (99.2–99.4)
	0.46 (−0.44; 1.35)	**LATG**	−0.41 (−1.39; 0.56)	−0.74 (−2.27; 0.78)	
	0.87 (0.21; 1.52)	0.41 (−0.56; 1.39)	**TLTG**	−0.33 (−1.5; 0.84)	
	1.20 (−0.14; 2.54)	0.74 (−0.78; 2.27)	0.33 (−0.84; 1.50)	**RTG**	
Time to first ambulation	**OTG**	−0.80 (−1.63; 0.03)	−0.81 (−1.52; −0.09)	−1.02 (−2.29; 0.26)	97.7 (97–98.3)
	0.80 (−0.03; 1.63)	**LATG**	−0.01 (−1.01; 0.99)	−0.22 (−1.67; 1.23)	
	0.81 (0.09; 1.01)	0.01 (−0.99; 1.01)	**TLTG**	−0.21 (−1.27; 0.85)	
	1.02 (−0.26; 2.29)	0.22 (−1.23; 1.67)	0.21 (−0.85; 1.27)	**RTG**	
LOS	**OTG**	−0.46 (−0.71; −0.21)	−0.55 (−0.77; −0.33)	−0.84 (−1.22; −0.45)	96.8 (96.4–97.2)
	0.46 (0.21; 0.71)	**LATG**	−0.09 (−0.39; 0.21)	−0.38 (−0.79; 0.03)	
	0.55 (0.33–0.77)	0.09 (−0.21; 0.39)	**TLTG**	−0.29 (−0.64; 0.06)	
	0.84 (0.45; 1.22)	0.38 (−0.03; 0.79)	0.29 (−0.06; 0.64)	**RTG**	

**Values in each column represent the relative effect of the referral treatment (bold) with the comparator.** Open total gastrectomy (**OTG**); lap-assisted total gastrectomy (**LATG**): totally laparoscopic total gastrectomy (**TLTG**); and robotic total gastrectomy (**RTG**); overall complications (**OC**); severe postoperative complications (**SPCs**); anastomotic leak (**AL**); surgical site infections (**SSI**); operating time (**OT**); hospital length of stay (**LOS**). Values are expressed as risk ratio (RR) and 95% credible intervals (95% CrIs). I^2^: heterogeneity.

## Data Availability

Data generated at a central, large-scale facility are available upon request from the corresponding author.

## Supplementary Materials

The following supporting information can be downloaded at https://www.mdpi.com/article/10.3390/cancers16193404/s1: Figure S1. Risk of bias for randomized controlled trials (RCT) assessed with use of the Cochrane risk-of-bias tool 2. Green circle: low risk of bias; red circle: high risk of bias; yellow circle: unclear risk of bias. Bias arising from the randomization process (R); bias due to deviations from intended interventions (D); bias due to missing outcome data (Mi); bias in measurement of the outcome (Me); bias in selection of the reported result (S); overall risk of bias (O). Table S1. Quality assessment of the included studies (ROBINS-I tool). Each domain is evaluated with one of the following: low, moderate, serious, critical, NI (no information). The categories of judgement for each study are low, moderate, serious, and critical risk of bias. File S1. PRISMA 2020 Checklist.

## Appendix A

Complete search strategy: (‘stomach cancer’/exp OR ‘cancer of the cardia’ OR ‘cancer of the gastric antrum’ OR ‘cancer of the gastric body’ OR ‘cancer of the gastric cardia’ OR ‘cancer of the gastric fundus’ OR ‘cancer, stomach’ OR ‘cardia cancer’ OR ‘gastric antral cancer’ OR ‘gastric antrum cancer’ OR ‘gastric body cancer’ OR ‘gastric cancer’ OR ‘gastric cardia cancer’ OR ‘gastric cardiac cancer’ OR ‘gastric malignancies’ OR ‘gastric malignancy’ OR ‘malignancies of the stomach’ OR ‘malignancy of the stomach’ OR ‘malignant gastric neoplasm’ OR ‘malignant gastric tumor’ OR ‘malignant neoplasm of the stomach’ OR ‘malignant neoplasms of the stomach’ OR ‘malignant tumor of the stomach’ OR ‘malignant tumors of the stomach’ OR ‘malignant tumour of the stomach’ OR ‘malignant tumours of the stomach’ OR ‘pyloric cancer’ OR ‘stomach cancer’ OR ‘stomach malignancies’ OR ‘stomach malignancy’) AND ((‘total gastrectomy’/exp OR ‘complete gastric resection’ OR ‘stomach total resection’ OR ‘total gastrectomy’ OR ‘total gastric resection’ OR ‘total stomach resection’) AND (‘open surgery’/exp OR ‘open surgery’ OR ‘open surgical repair’)) AND ((‘laparoscopic total gastrectomy’/exp OR ‘laparoscopic assisted total gastrectomy’ OR ‘laparoscopic total gastrectomy’ OR ‘laparoscopyassisted total gastrectomy’ OR ‘total laparoscopic gastrectomy’ OR ‘total laparoscopic-assisted gastrectomy’) OR (‘robot assisted surgery’/exp OR ‘robot aided surgery’ OR ‘robot assisted surgery’ OR ‘robot surgery’ OR ‘robotic aided surgery’ OR ‘robotic surgery’ OR ‘robotic surgical procedure’ OR ‘robotic surgical procedures’ OR ‘robotically assisted surgery’)) AND ((‘postoperative complication’/exp OR ‘complication after operation’ OR ‘complication after surgery’ OR ‘complication, postoperative’ OR ‘complication, surgical’ OR ‘post-operation complication’ OR ‘post-operative complication’ OR ‘post-operative complications’ OR ‘post-surgery complication’ OR ‘post-surgical complication’ OR ‘postoperation complication’ OR ‘postoperative complication’ OR ‘postoperative complications’ OR ‘postsurgery complication’ OR ‘postsurgical complication’ OR ‘surgery complication’ OR ‘surgery complications’ OR ‘surgery-associated complication’ OR ‘surgery-derived complication’ OR ‘surgery-induced complication’ OR ‘surgery-related complication’ OR ‘surgical complication’) OR (‘anastomosis complication’/exp OR ‘anastomosis complication’ OR ‘anastomotic complication’) OR ‘short term outcome’/exp OR (bleeding/exp OR ‘abnormal bleeding’ OR ‘bleeding’ OR ‘bleeding complication’ OR ‘blood effusion’ OR ‘blood loss’ OR ‘capillary bleeding’ OR ‘haemorrhage’ OR ‘haemorrhage model’ OR ‘haemorrhagic activity’ OR ‘haemorrhaging’ OR ‘hemorrhage’ OR ‘hemorrhage model’ OR ‘hemorrhagia’ OR ‘hemorrhagic activity’ OR ‘hemorrhaging’ OR ‘spontaneous haemorrhage’ OR ‘spontaneous hemorrhage’) OR ‘duodenal stump leakage’/exp OR ‘duodenal stump leak’/exp OR ‘duodenal stump fistula’/exp OR (‘acute pancreatitis’/exp OR ‘acute pancreatitis’ OR ‘pancreatitis acuta’) OR ‘pancreatic leak’/exp OR (‘pancreas fistula’/exp OR ‘pancreas fistula’ OR ‘pancreatic fistula’) OR ‘pancreatic leakage’/exp OR (‘lung complication’/exp OR ‘complication, lung’ OR ‘lung complication’ OR ‘pulmonary complication’) OR (‘surgical infection’/exp OR ‘SSI (surgical site infection)’ OR ‘infected surgical site’ OR ‘infected surgical wound’ OR ‘infection at the operative site’ OR ‘infection at the surgery site’ OR ‘infection at the surgical site’ OR ‘infection of the operative site’ OR ‘infection of the surgery site’ OR ‘infection of the surgical site’ OR ‘infection of the surgical wound’ OR ‘infection, surgical’ OR ‘operative site infection’ OR ‘post-operative wound infection’ OR ‘postoperative wound infections’ OR ‘postoperative wound infection’ OR ‘postoperative wound infections’ OR ‘surgery site infection’ OR ‘surgical infection’ OR ‘surgical infections’ OR ‘surgical site infection’ OR ‘surgical wound infection’ OR ‘surgical wound infections’) OR (thrombosis/exp OR ‘acute thrombosis’ OR ‘arm thrombosis’ OR ‘atherothrombosis’ OR ‘rethrombosis’ OR ‘sclerothrombosis’ OR ‘thrombo-obliterative disease’ OR ‘thrombo-occlusive disease’ OR ‘thromboocclusive disorder’ OR ‘thromboobliterative disease’ OR ‘thromboocclusive disease’ OR ‘thromboocclusive disorder’ OR ‘thrombosis’ OR ‘thrombosis induction’ OR ‘thrombotic disease’ OR ‘thrombotic disorder’ OR ‘thrombotic occlusion’)).

## References

[1] Sung H., Ferlay J., Siegel R.L., Laversanne M., Soerjomataram I., Jemal A., Bray F. (2021). Global Cancer Statistics 2020: GLOBOCAN Estimates of Incidence and Mortality Worldwide for 36 Cancers in 185 Countries. CA Cancer J. Clin..

[2] Lordick F., Carneiro F., Cascinu S., Fleitas T., Haustermans K., Piessen G., Vogel A., Smyth E.C. (2022). Gastric Cancer: ESMO Clinical Practice Guideline for Diagnosis, Treatment and Follow-Up. Ann. Oncol..

[3] Japanese Gastric Cancer Association (2023). Japanese Gastric Cancer Treatment Guidelines 2021 (6th Edition). Gastric Cancer.

[4] Lei X., Wang Y., Shan F., Li S., Jia Y., Miao R., Xue K., Li Z., Ji J., Li Z. (2022). Short-and Long-Term Outcomes of Laparoscopic versus Open Gastrectomy in Patients with Gastric Cancer: A Systematic Review and Meta-Analysis of Randomized Controlled Trials. World J. Surg. Oncol..

[5] Kitano S., Iso Y., Moriyama M., Sugimachi K. (1994). Laparoscopy-Assisted Billroth I Gastrectomy. Surg. Laparosc. Endosc..

[6] Hashizume M., Shimada M., Tomikawa M., Ikeda Y., Takahashi I., Abe R., Koga F., Gotoh N., Konishi K., Maehara S. (2002). Early Experiences of Endoscopic Procedures in General Surgery Assisted by a Computer-Enhanced Surgical System. Surg. Endosc..

[7] Makuuchi R., Kamiya S., Tanizawa Y., Bando E., Terashima M. (2019). Robotic Surgery for Gastric Cancer. Mini-Invasive Surg..

[8] Marohn M.R., Hanly E.J. (2004). Twenty-First Century Surgery Using Twenty-First Century Technology: Surgical Robotics. Curr. Surg..

[9] Davey M.G., Temperley H.C., O’Sullivan N.J., Marcelino V., Ryan O.K., Ryan É.J., Donlon N.E., Johnston S.M., Robb W.B. (2023). Minimally Invasive and Open Gastrectomy for Gastric Cancer: A Systematic Review and Network Meta-Analysis of Randomized Clinical Trials. Ann. Surg. Oncol..

[10] Ferrari D., Violante T., Novelli M., Starlinger P.P., Smoot R.L., Reisenauer J.S., Larson D.W. (2024). The Death of Laparoscopy. Surg. Endosc..

[11] Watt J., Del Giovane C. (2022). Network Meta-Analysis. Methods Mol. Biol..

[12] Caldwell D.M., Ades A.E., Higgins J.P.T. (2005). Simultaneous Comparison of Multiple Treatments: Combining Direct and Indirect Evidence. BMJ.

[13] Page M.J., McKenzie J.E., Bossuyt P.M., Boutron I., Hoffmann T.C., Mulrow C.D., Shamseer L., Tetzlaff J.M., Akl E.A., Brennan S.E. (2021). The PRISMA 2020 Statement: An Updated Guideline for Reporting Systematic Reviews. BMJ.

[14] Goossen K., Tenckhoff S., Probst P., Grummich K., Mihaljevic A.L., Büchler M.W., Diener M.K. (2018). Optimal Literature Search for Systematic Reviews in Surgery. Langenbecks Arch. Surg..

[15] Dindo D., Demartines N., Clavien P.A. (2004). Classification of Surgical Complications: A New Proposal with Evaluation in a Cohort of 6336 Patients and Results of a Survey. Ann. Surg..

[16] Sterne J.A., Hernán M.A., Reeves B.C., Savović J., Berkman N.D., Viswanathan M., Henry D., Altman D.G., Ansari M.T., Boutron I. (2016). ROBINS-I: A Tool for Assessing Risk of Bias in Non-Randomised Studies of Interventions. BMJ.

[17] Higgins J.P.T., Altman D.G., Gøtzsche P.C., Jüni P., Moher D., Oxman A.D., Savović J., Schulz K.F., Weeks L., Sterne J.A.C. (2011). The Cochrane Collaboration’s Tool for Assessing Risk of Bias in Randomised Trials. BMJ.

[18] Salanti G., Higgins J.P.T., Ades A.E., Ioannidis J.P.A. (2008). Evaluation of Networks of Randomized Trials. Stat. Methods Med. Res..

[19] Jackson D., White I.R., Riley R.D. (2013). A Matrix-Based Method of Moments for Fitting the Multivariate Random Effects Model for Meta-Analysis and Meta-Regression. Biom. J..

[20] Higgins J.P.T., Thompson S.G. (2002). Quantifying Heterogeneity in a Meta-Analysis. Stat. Med..

[21] Salanti G. (2012). Indirect and Mixed-Treatment Comparison, Network, or Multiple-Treatments Meta-Analysis: Many Names, Many Benefits, Many Concerns for the next Generation Evidence Synthesis Tool. Res. Synth. Methods.

[22] Nikolakopoulou A., Higgins J.P.T., Papakonstantinou T., Chaimani A., Del Giovane C., Egger M., Salanti G. (2020). CINeMA: An Approach for Assessing Confidence in the Results of a Network Meta-Analysis. PLoS Med..

[23] Balduzzi S., Rücker G., Nikolakopoulou A., Papakonstantinou T., Salanti G., Efthimiou O., Schwarzer G. (2023). Netmeta: An R Package for Network Meta-Analysis Using Frequentist Methods. J. Stat. Softw..

[24] Eom S.S., Park S.H., Eom B.W., Yoon H.M., Kim Y.W., Ryu K.W. (2022). Short and Long-Term Surgical Outcomes of Laparoscopic Total Gastrectomy Compared with Open Total Gastrectomy in Gastric Cancer Patients. Cancers.

[25] Illuminati G., D’Urso A., Fiori E., Cerasari S., Nardi P., Lapergola A., Pasqua R., Sorrenti S., Pironi D., Lauro A. (2023). Laparoscopy-Assisted vs Open Total Gastrectomy with D2 Lymphadenectomy for Advanced Gastric Cancer: Results of a Retrospective, Multicenter Study. Updates Surg..

[26] Jia Z., Cao S., Meng C., Liu X., Li Z., Tian Y., Yu J., Sun Y., Xu J., Liu G. (2023). Intraoperative Performance and Outcomes of Robotic and Laparoscopic Total Gastrectomy for Gastric Cancer: A High-Volume Center Retrospective Propensity Score Matching Study. Cancer Med..

[27] Kinoshita T., Akimoto E., Yura M., Yoshida M. (2022). Survival Outcomes of Laparoscopic versus Open Total Gastrectomy with Nodal Dissection for Gastric Cancer in a High-Volume Japanese Center: A Propensity Score-Matched Analysis. Ann. Gastroenterol. Surg..

[28] Salvador-Rosés H., Escartín A., Muriel P., Santamaría M., González M., Jara J., Vela F., Olsina J.J. (2023). Robotic versus Open Approach in Total Gastrectomy for Gastric Cancer: A Comparative Single-Center Study of Perioperative Outcomes. J. Robot. Surg..

[29] Zheng H.-L., Shen L.-l., Xu B.-b., Chen Q.-Y., Lu J., Xue Z., Jia-Lin, Xie J.-W., Li P., Huang C.-M. (2023). Oncological Outcomes of Laparoscopic versus Open Radical Total Gastrectomy for Upper-Middle Gastric Cancer after Neoadjuvant Chemotherapy: A Study of Real-World Data. Surg. Endosc..

[30] Hu H.-T., Ma F.-H., Xiong J.-P., Li Y., Jin P., Liu H., Ma S., Kang W.-Z., Tian Y.-T. (2022). Laparoscopic vs Open Total Gastrectomy for Advanced Gastric Cancer Following Neoadjuvant Therapy: A Propensity Score Matching Analysis. World J. Gastrointest. Surg..

[31] Hikage M., Fujiya K., Kamiya S., Tanizawa Y., Bando E., Terashima M. (2022). Comparisons of Surgical Outcomes between Robotic and Laparoscopic Total Gastrectomy in Patients with Clinical Stage I/IIA Gastric Cancer. Surg. Endosc..

[32] Chen Q.Y., Zhong Q., Liu Z.Y., Li P., Wang J.B., Lin J.X., Lu J., Cao L.L., Lin M., Tu R.H. (2022). Surgical Outcomes, Technical Performance, and Surgery Burden of Robotic Total Gastrectomy for Locally Advanced Gastric Cancer: A Prospective Study. Ann. Surg..

[33] Cui H., Zhang K.-C., Cao B., Deng H., Liu G.-B., Song L.-Q., Zhao R.-Y., Liu Y., Chen L., Wei B. (2022). Short and Long-Term Outcomes between Laparoscopic and Open Total Gastrectomy for Advanced Gastric Cancer after Neoadjuvant Chemotherapy. World J. Gastrointest. Surg..

[34] Di Carlo S., Siragusa L., Fassari A., Fiori E., La Rovere F., Izzo P., Usai V., Cavallaro G., Franceschilli M., Dhimolea S. (2022). Laparoscopic versus Open Total Gastrectomy for Locally Advanced Gastric Cancer: Short and Long-Term Results. Curr. Oncol..

[35] Li Z., Qian F., Zhao Y., Chen J., Zhang F., Li Z., Wang X., Li P., Liu J., Wen Y. (2022). A Comparative Study on Perioperative Outcomes between Robotic versus Laparoscopic D2 Total Gastrectomy. Int. J. Surg..

[36] Lin G.T., Chen J.Y., Chen Q.Y., Que S.J., Liu Z.Y., Zhong Q., Wang J.B., Lin J.X., Lu J., Lin M. (2023). Patient-Reported Outcomes of Individuals with Gastric Cancer Undergoing Totally Laparoscopic Versus Laparoscopic-Assisted Total Gastrectomy: A Real-World, Propensity Score-Matching Analysis. Ann. Surg. Oncol..

[37] Qiu X.T., Zheng C.Y., Liang Y.L., Zheng L.Z., Zu B., Chen H.H., Dong Z.Y., Zhu L.M., Lin W. (2022). Totally Laparoscopic Total Gastrectomy Using the “Enjoyable Space” Approach Coupled with Self-Pulling and Latter Transection Reconstruction versus Laparoscopic-Assisted Total Gastrectomy for Upper Gastric Cancer: Short-Term Outcomes. Wideochir. Inne Tech. Maloinwazyjne.

[38] Shibasaki S., Nakauchi M., Serizawa A., Nakamura K., Akimoto S., Tanaka T., Inaba K., Uyama I., Suda K. (2022). Clinical Advantage of Standardized Robotic Total Gastrectomy for Gastric Cancer: A Single-Center Retrospective Cohort Study Using Propensity-Score Matching Analysis. Gastric Cancer.

[39] Wang Z.K., Lin J.X., Wang F.H., Xie J.W., Wang J.B., Lu J., Chen Q.Y., Cao L.L., Lin M., Tu R.H. (2022). Robotic Spleen-Preserving Total Gastrectomy Shows Better Short-Term Advantages: A Comparative Study with Laparoscopic Surgery. Surg. Endosc..

[40] van der Wielen N., Straatman J., Daams F., Rosati R., Parise P., Weitz J., Reissfelder C., Diez del Val I., Loureiro C., Parada-González P. (2021). Open versus Minimally Invasive Total Gastrectomy after Neoadjuvant Chemotherapy: Results of a European Randomized Trial. Gastric Cancer.

[41] Challine A., Voron T., Dousset B., Creavin B., Katsahian S., Parc Y., Lazzati A., Lefèvre J.H. (2021). Postoperative Outcomes after Laparoscopic or Open Gastrectomy. A National Cohort Study of 10,343 Patients. Eur. J. Surg. Oncol..

[42] Fan Y., Liu M., Li S., Yu J., Qi X., Tan F., Xu K., Zhang N., Yao Z., Yang H. (2021). Surgical and Oncological Efficacy of Laparoscopic-Assisted Total Gastrectomy versus Open Total Gastrectomy for Gastric Cancer by Propensity Score Matching: A Retrospective Comparative Study. J. Cancer Res. Clin. Oncol..

[43] Feng X., Chen X., Ye Z., Xiong W., Yao X., Wang W., Wang J., Chen L., Li Y. (2021). Laparoscopic Versus Open Total Gastrectomy for Advanced Gastric Cancer: A Multicenter, Propensity Score-Matched Cohort Study in China. Front. Oncol..

[44] Ko C.S., Choi N.R., Kim B.S., Yook J.H., Kim M.J., Kim B.S. (2021). Totally Laparoscopic Total Gastrectomy Using the Modified Overlap Method and Conventional Open Total Gastrectomy: A Comparative Study. World J. Gastroenterol..

[45] Kumamoto T., Ishida Y., Igeta M., Hojo Y., Nakamura T., Kurahashi Y., Shinohara H. (2022). Potential Advantages of Robotic Total Gastrectomy for Gastric Cancer: A Retrospective Comparative Cohort Study. J. Robot. Surg..

[46] Roh C.K., Lee S., Son S.Y., Hur H., Han S.U. (2021). Textbook Outcome and Survival of Robotic versus Laparoscopic Total Gastrectomy for Gastric Cancer: A Propensity Score Matched Cohort Study. Sci. Rep..

[47] Wang Y., Lei X., Liu Z., Shan F., Ying X., Li Z., Ji J. (2021). Short-Term Outcomes of Laparoscopic versus Open Total Gastrectomy after Neoadjuvant Chemotherapy: A Cohort Study Using the Propensity Score Matching Method. J. Gastrointest. Oncol..

[48] Liu F., Huang C., Xu Z., Su X., Zhao G., Ye J., Du X., Huang H., Hu J., Li G. (2020). Morbidity and Mortality of Laparoscopic vs Open Total Gastrectomy for Clinical Stage I Gastric Cancer: The CLASS02 Multicenter Randomized Clinical Trial. JAMA Oncol..

[49] Komatsu S., Kosuga T., Kubota T., Okamoto K., Konishi H., Shiozaki A., Fujiwara H., Ichikawa D., Otsuji E. (2020). Comparison of Short- and Long-Term Outcomes Following Laparoscopy and Open Total Gastrectomy for Gastric Cancer: A Propensity Score-Matched Analysis. Am. J. Transl. Res..

[50] Yang C., Shi Y., Xie S., Chen J., Zhao Y., Qian F., Hao Y., Tang B., Yu P. (2020). Short-Term Outcomes of Robotic- versus Laparoscopic-Assisted Total Gastrectomy for Advanced Gastric Cancer: A Propensity Score Matching Study. BMC Cancer.

[51] Lee H., Kim W., Lee J. (2020). Long-Term Outcomes of Laparoscopic versus Open Total Gastrectomy for Advanced Gastric Cancer: A Propensity Score-Matched Analysis. Dig. Surg..

[52] Sakamoto T., Fujiogi M., Matsui H., Fushimi K., Yasunaga H. (2020). Short-Term Outcomes of Laparoscopic and Open Total Gastrectomy for Gastric Cancer: A Nationwide Retrospective Cohort Analysis. Ann. Surg. Oncol..

[53] Zhao Y., Zhang J., Yang D., Tang Z., Wang Q. (2019). Feasibility of Laparoscopic Total Gastrectomy for Advanced Siewert Type I and Type III Esophagogastric Junction Carcinoma: A Propensity Score-Matched Case-Control Study. Asian J. Surg..

[54] Ye S.P., Shi J., Liu D.N., Jiang Q.G., Lei X., Qiu H., Li T.Y. (2019). Robotic-Assisted versus Conventional Laparoscopic-Assisted Total Gastrectomy with D2 Lymphadenectomy for Advanced Gastric Cancer: Short-Term Outcomes at a Mono-Institution. BMC Surg..

[55] Aoyama T., Yoshikawa T., Maezawa Y., Kano K., Hara K., Sato T., Hayashi T., Yamada T., Cho H., Ogata T. (2018). A Comparison of the Body Composition Changes between Laparoscopy-Assisted and Open Total Gastrectomy for Gastric Cancer. Vivo.

[56] Li Z., Liu Y., Bai B., Yu D., Lian B., Zhao Q. (2019). Surgical and Long-Term Survival Outcomes after Laparoscopic and Open Total Gastrectomy for Locally Advanced Gastric Cancer: A Propensity Score-Matched Analysis. World J. Surg..

[57] Wang J., Wang J.C., Song B., Dai X.D., Zhang X.Y. (2019). Comparative Study of Laparoscopic-Assisted and Open Total Gastrectomy for Siewert Types II and III Adenocarcinoma of the Esophagogastric Junction. J. Cell. Physiol..

[58] Etoh T., Honda M., Kumamaru H., Miyata H., Yoshida K., Kodera Y., Kakeji Y., Inomata M., Konno H., Seto Y. (2018). Morbidity and Mortality from a Propensity Score-Matched, Prospective Cohort Study of Laparoscopic versus Open Total Gastrectomy for Gastric Cancer: Data from a Nationwide Web-Based Database. Surg. Endosc..

[59] Chen K., Pan Y., Zhai S.T., Yu W.H., Pan J.H., Zhu Y.P., Chen Q.L., Wang X.F. (2017). Totally Laparoscopic versus Open Total Gastrectomy for Gastric Cancer: A Case-Matched Study about Short-Term Outcomes. Medicine.

[60] Chen X.Z., Wang S.Y., Wang Y.S., Jiang Z.H., Zhang W.H., Liu K., Yang K., Chen X.L., Zhao L.Y., Qiu M. (2017). Comparisons of Short-Term and Survival Outcomes of Laparoscopy-Assisted versus Open Total Gastrectomy for Gastric Cancer Patients. Oncotarget.

[61] Lin J.X., Lin J.L., Zheng C.H., Li P., Xie J.W., Wang J.B., Lu J., Chen Q.Y., Cao L.L., Lin M. (2017). Short- and Long-Term Outcomes of Laparoscopy-Assisted versus Open Total Gastrectomy for Gastric Cancer: A Propensity Score-Matched Analysis. Oncotarget.

[62] Kim E.Y., Choi H.J., Cho J.B., Lee J. (2016). Totally Laparoscopic Total Gastrectomy versus Laparoscopically Assisted Total Gastrectomy for Gastric Cancer. Anticancer Res..

[63] Kim H.B., Kim S.M., Ha M.H., Seo J.E., Choi M.G., Sohn T.S., Bae J.M., Kim S., Lee J.H. (2016). Comparison of Reduced Port Totally Laparoscopic-Assisted Total Gastrectomy (Duet TLTG) and Conventional Laparoscopic-Assisted Total Gastrectomy. Surg. Laparosc. Endosc. Percutan. Tech..

[64] Wu H., Li W., Chen G., Wang W., Zheng Z., Li J., Li X. (2016). Outcome of Laparoscopic Total Gastrectomy for Gastric Carcinoma. J. BUON.

[65] Shu B., Lei S., Li F., Hua S., Chen Y., Huo Z. (2016). Laparoscopic Total Gastrectomy Compared with Open Resection for Gastric Carcinoma: A Case-Matched Study with Long-Term Follow-Up. J. BUON.

[66] Lu Y., Jiang B., Liu T. (2016). Laparoscopic versus Open Total Gastrectomy for Advanced Proximal Gastric Carcinoma: A Matched Pair Analysis. J. BUON.

[67] Huang C.M., Lv C.B., Lin J.X., Chen Q.Y., Zheng C.H., Li P., Xie J.W., Wang J.B., Lu J., Cao L.L. (2017). Laparoscopic-Assisted versus Open Total Gastrectomy for Siewert Type II and III Esophagogastric Junction Carcinoma: A Propensity Score-Matched Case-Control Study. Surg. Endosc..

[68] Park J.M., Kim H.I., Han S.U., Yang H.K., Kim Y.W., Lee H.J., An J.Y., Kim M.C., Park S., Song K.Y. (2016). Who May Benefit from Robotic Gastrectomy?: A Subgroup Analysis of Multicenter Prospective Comparative Study Data on Robotic versus Laparoscopic Gastrectomy. Eur. J. Surg. Oncol..

[69] Shida A., Mitsumori N., Fujioka S., Takano Y., Iwasaki T., Takahashi N., Ishibashi Y., Omura N., Yanaga K. (2016). Comparison of Short-Term and Long-Term Clinical Outcomes between Laparoscopic and Open Total Gastrectomy for Patients with Gastric Cancer. Surg. Laparosc. Endosc. Percutan. Tech..

[70] Zhang G.T., Zhang X.D., Xue H.Z. (2017). Open Versus Hand-Assisted Laparoscopic Total Gastric Resection with D2 Lymph Node Dissection for Adenocarcinoma: A Case-Control Study. Surg. Laparosc. Endosc. Percutan. Tech..

[71] Ramagem C.A.G., Linhares M., Lacerda C.F., Bertulucci P.A., Wonrath D., de Oliveira A.T.T. (2015). Comparison of Laparoscopic Total Gastrectomy and Laparotomic Total Gastrectomy for Gastric Cancer. Arq. Bras. Cir. Dig..

[72] Lee J.H., Nam B.H., Ryu K.W., Ryu S.Y., Park Y.K., Kim S., Kim Y.W. (2015). Comparison of Outcomes after Laparoscopy-Assisted and Open Total Gastrectomy for Early Gastric Cancer. Br. J. Surg..

[73] Lu J., Huang C.M., Zheng C.H., Li P., Xie J.W., Wang J.B., Lin J.X., Chen Q.Y., Cao L.L., Lin M. (2015). Short- and Long-Term Outcomes after Laparoscopic versus Open Total Gastrectomy for Elderly Gastric Cancer Patients: A Propensity Score-Matched Analysis. J. Gastrointest. Surg..

[74] Shen W., Xi H., Wei B., Cui J., Bian S., Zhang K., Wang N., Huang X., Chen L. (2016). Robotic versus Laparoscopic Gastrectomy for Gastric Cancer: Comparison of Short-Term Surgical Outcomes. Surg. Endosc..

[75] Song J.H., Choi Y.Y., An J.Y., Kim D.W., Hyung W.J., Noh S.H. (2015). Short-Term Outcomes of Laparoscopic Total Gastrectomy Performed by a Single Surgeon Experienced in Open Gastrectomy: Review of Initial Experience. J. Gastric Cancer.

[76] Lee S.R., Kim H.O., Son B.H., Shin J.H., Yoo C.H. (2014). Laparoscopic-Assisted Total Gastrectomy versus Open Total Gastrectomy for Upper and Middle Gastric Cancer in Short-Term and Long-Term Outcomes. Surg. Laparosc. Endosc. Percutan. Tech..

[77] Lee M.S., Lee J.H., Park D.J., Lee H.J., Kim H.H., Yang H.K. (2013). Comparison of Short- and Long-Term Outcomes of Laparoscopic-Assisted Total Gastrectomy and Open Total Gastrectomy in Gastric Cancer Patients. Surg. Endosc..

[78] Bo T., Peiwu Y., Feng Q., Yongliang Z., Yan S., Yingxue H., Huaxing L. (2013). Laparoscopy-Assisted vs. Open Total Gastrectomy for Advanced Gastric Cancer: Long-Term Outcomes and Technical Aspects of a Case-Control Study. J. Gastrointest. Surg..

[79] Guan G., Jiang W., Chen Z., Liu X., Lu H., Zhang X. (2013). Early Results of a Modified Splenic Hilar Lymphadenectomy in Laparoscopy-Assisted Total Gastrectomy for Gastric Cancer with Stage CT1-2: A Case-Control Study. Surg Endosc.

[80] Kim H.S., Kim B.S., Lee I.S., Lee S., Yook J.H., Kim B.S. (2013). Comparison of Totally Laparoscopic Total Gastrectomy and Open Total Gastrectomy for Gastric Cancer. J. Laparoendosc. Adv. Surg. Tech..

[81] Kim K.H., Kim Y.M., Kim M.C., Jung G.J. (2014). Is Laparoscopy-Assisted Total Gastrectomy Feasible for the Treatment of Gastric Cancer? A Case-Matched Study. Dig. Surg..

[82] Jeong O., Jung M.R., Kim G.Y., Kim H.S., Ryu S.Y., Park Y.K. (2013). Comparison of Short-Term Surgical Outcomes between Laparoscopic and Open Total Gastrectomy for Gastric Carcinoma: Case-Control Study Using Propensity Score Matching Method. J. Am. Coll. Surg..

[83] Hong L., Han Y., Jin Y., Zhang H., Zhao Q. (2013). The Short-Term Outcome in Esophagogastric Junctional Adenocarcinoma Patients Receiving Total Gastrectomy: Laparoscopic versus Open Gastrectomy—A Retrospective Cohort Study. Int. J. Surg..

[84] Eom B.W., Kim Y.W., Lee S.E., Ryu K.W., Lee J.H., Yoon H.M., Cho S.J., Kook M.C., Kim S.J. (2012). Survival and Surgical Outcomes after Laparoscopy-Assisted Total Gastrectomy for Gastric Cancer: Case-Control Study. Surg. Endosc..

[85] Kim M.G., Kim B.S., Kim T.H., Kim K.C., Yook J.H., Kim B.S. (2011). The Effects of Laparoscopic Assisted Total Gastrectomy on Surgical Outcomes in the Treatment of Gastric Cancer. J. Korean Surg. Soc..

[86] Yoon H.M., Kim Y.W., Lee J.H., Ryu K.W., Eom B.W., Park J.Y., Choi I.J., Kim C.G., Lee J.Y., Cho S.J. (2012). Robot-Assisted Total Gastrectomy Is Comparable with Laparoscopically Assisted Total Gastrectomy for Early Gastric Cancer. Surg. Endosc..

[87] Sakuramoto S., Kikuchi S., Futawatari N., Katada N., Moriya H., Hirai K., Yamashita K., Watanabe M. (2009). Laparoscopy-Assisted Pancreas- and Spleen-Preserving Total Gastrectomy for Gastric Cancer as Compared with Open Total Gastrectomy. Surg. Endosc..

[88] Kawamura H., Yokota R., Homma S., Kondo Y. (2009). Comparison of Invasiveness between Laparoscopy-Assisted Total Gastrectomy and Open Total Gastrectomy. World J. Surg..

[89] Mochiki E., Toyomasu Y., Ogata K., Andoh H., Ohno T., Aihara R., Asao T., Kuwano H. (2008). Laparoscopically Assisted Total Gastrectomy with Lymph Node Dissection for Upper and Middle Gastric Cancer. Surg. Endosc. Other Interv. Tech..

[90] Topal B., Leys E., Ectors N., Aerts R., Penninckx F. (2008). Determinants of Complications and Adequacy of Surgical Resection in Laparoscopic versus Open Total Gastrectomy for Adenocarcinoma. Surg. Endosc. Other Interv. Tech..

[91] Dulucq J.L., Wintringer P., Stabilini C., Solinas L., Perissat J., Mahajna A. (2005). Laparoscopic and Open Gastric Resections for Malignant Lesions: A Prospective Comparative Study. Surg. Endosc. Other Interv. Tech..

[92] Guan W.L., He Y., Xu R.H. (2023). Gastric Cancer Treatment: Recent Progress and Future Perspectives. J. Hematol. Oncol..

[93] Ajani J.A., D’Amico T.A., Bentrem D.J., Chao J., Cooke D., Corvera C., Das P., Enzinger P.C., Enzler T., Fanta P. (2022). Gastric Cancer, Version 2.2022, NCCN Clinical Practice Guidelines in Oncology. J. Natl. Compr. Cancer Netw..

[94] Rogers J.E., Ajani J.A. (2023). Recent Advances in the Management of Gastric Adenocarcinoma Patients. Fac. Rev..

[95] Aiolfi A., Lombardo F., Matsushima K., Sozzi A., Cavalli M., Panizzo V., Bonitta G., Bona D. (2021). Systematic Review and Updated Network Meta-Analysis of Randomized Controlled Trials Comparing Open, Laparoscopic-Assisted, and Robotic Distal Gastrectomy for Early and Locally Advanced Gastric Cancer. Surgery.

[96] Hyung W.J., Yang H.K., Han S.U., Lee Y.J., Park J.M., Kim J.J., Kwon O.K., Kong S.H., Kim H.I., Lee H.J. (2019). A Feasibility Study of Laparoscopic Total Gastrectomy for Clinical Stage I Gastric Cancer: A Prospective Multi-Center Phase II Clinical Trial, KLASS 03. Gastric Cancer.

[97] Kunisaki C., Katai H., Sakuramoto S., Mizusawa J., Katayama H., Kadoya S., Yamada T., Kinoshita T., Yoshikawa T., Terashima M. (2024). A Nonrandomized Controlled Trial: Long-Term Outcomes of LATG/LAPG for CStage I Gastric Cancer: Japan Clinical Oncology Group Study JCOG1401. Gastric Cancer.

[98] Wu Q., Wang Y., Peng Q., Bai M., Shang Z., Li L., Tian F., Jing C. (2024). Safety and Effectiveness of Totally Laparoscopic Total Gastrectomy vs Laparoscopic-Assisted Total Gastrectomy: A Meta-Analysis. Int. J. Surg..

[99] Vasavada B., Patel H. (2021). Laparoscopic vs Open Gastrectomy: An Updated Meta-Analysis of Randomized Control Trials for Short-Term Outcomes. Indian J. Surg. Oncol..

[100] Wei Y., Yu D., Li Y., Fan C., Li G. (2018). Laparoscopic versus Open Gastrectomy for Advanced Gastric Cancer: A Meta-Analysis Based on High-Quality Retrospective Studies and Clinical Randomized Trials. Clin. Res. Hepatol. Gastroenterol..

[101] Eom B.W., Ahn H.S., Lee I.S., Min J.S., Son Y.G., Lee S.E., Kim J.H., Lee S.Y., Kim J.H., Ahn S.H. (2016). Korean Gastric Cancer Association Nationwide Survey on Gastric Cancer in 2014. J. Gastric Cancer.

[102] Ryu S.W., Eom B.W., Kim D.J., Huh Y.J., Jeong S.H., Suh Y.S., Kim J.W., Kwon I.G., Kwon O.K., Lee I.S. (2021). Korean Gastric Cancer Association-Led Nationwide Survey on Surgically Treated Gastric Cancers in 2019. J. Gastric Cancer.

[103] Park S.H., Han M., Yoon H.M., Ryu K.W., Kim Y.W., Eom B.W. (2024). Real-World Nationwide Outcomes of Minimally Invasive Surgery for Advanced Gastric Cancer Based on Korean Gastric Cancer Association-Led Survey. J. Gastric Cancer.

[104] Manara M., Aiolfi A., Sozzi A., Calì M., Grasso F., Rausa E., Bonitta G., Bonavina L., Bona D. (2024). Short-Term Outcomes Analysis Comparing Open, Laparoscopic, Laparoscopic-Assisted, and Robotic Distal Gastrectomy for Locally Advanced Gastric Cancer: A Randomized Trials Network Analysis. Cancers.

[105] Etoh T., Ohyama T., Sakuramoto S., Tsuji T., Lee S.W., Yoshida K., Koeda K., Hiki N., Kunisaki C., Tokunaga M. (2023). Five-Year Survival Outcomes of Laparoscopy-Assisted vs Open Distal Gastrectomy for Advanced Gastric Cancer: The JLSSG0901 Randomized Clinical Trial. JAMA Surg..

[106] Wang W.-J., Li H.-T., Yu J.-P., Su L., Guo C.-A., Chen P., Yan L., Li K., Ma Y.-W., Wang L. (2019). Severity and Incidence of Complications Assessed by the Clavien–Dindo Classification Following Robotic and Laparoscopic Gastrectomy for Advanced Gastric Cancer: A Retrospective and Propensity Score-Matched Study. Surg. Endosc..

[107] Aiolfi A., Sozzi A., Bonitta G., Lombardo F., Cavalli M., Campanelli G., Bonavina L., Bona D. (2023). Short-Term Outcomes of Different Esophagojejunal Anastomotic Techniques during Laparoscopic Total Gastrectomy: A Network Meta-Analysis. Surg. Endosc..

[108] Yang Y., Chen Y., Hu Y., Feng Y., Mao Q., Xue W. (2022). Outcomes of Laparoscopic versus Open Total Gastrectomy with D2 Lymphadenectomy for Gastric Cancer: A Systematic Review and Meta-Analysis. Eur. J. Med. Res..

[109] Sozzi A., Aiolfi A., Matsushima K., Bonitta G., Lombardo F., Viti M., Russo A., Campanelli G., Bona D. (2023). Linear- Versus Circular-Stapled Esophagojejunostomy during Total Gastrectomy: Systematic Review and Meta-Analysis. J. Laparoendosc. Adv. Surg. Tech..

[110] Garbarino G.M., Laracca G.G., Lucarini A., Piccolino G., Mercantini P., Costa A., Tonini G., Canali G., Muttillo E.M., Costa G. (2022). Laparoscopic versus Open Surgery for Gastric Cancer in Western Countries: A Systematic Review and Meta-Analysis of Short- and Long-Term Outcomes. J. Clin. Med..

[111] Trastulli S., Desiderio J., Lin J.X., Reim D., Zheng C.H., Borghi F., Cianchi F., Norero E., Nguyen N.T., Qi F. (2023). Open vs Robotic Gastrectomy with D2 Lymphadenectomy: A Propensity Score-Matched Analysis on 1469 Patients from the IMIGASTRIC Prospective Database. Langenbecks Arch. Surg..

[112] Liu H., Kinoshita T., Tonouchi A., Kaito A., Tokunaga M. (2019). What Are the Reasons for a Longer Operation Time in Robotic Gastrectomy than in Laparoscopic Gastrectomy for Stomach Cancer?. Surg. Endosc..

[113] Omori T., Yamamoto K., Hara H., Shinno N., Yamamoto M., Fujita K., Kanemura T., Takeoka T., Akita H., Wada H. (2022). Comparison of Robotic Gastrectomy and Laparoscopic Gastrectomy for Gastric Cancer: A Propensity Score-Matched Analysis. Surg. Endosc..

[114] Wang X., Yao Y., Qian H., Li H., Zhu X. (2019). Longer Operating Time During Gastrectomy Has Adverse Effects on Short-Term Surgical Outcomes. J. Surg. Res..

[115] Park S.-H., Shin Y.-R., Hur H., Lee C.M., Min J.S., Ryu S.W., Chae H.D., Jeong O., Choi C.-I., Song K.-Y. (2023). Exploring Ideal Operative Time for Best Outcomes in Gastric Cancer Surgery: A Multi-Institutional Study Based on KLASS-07 Database. Chin. J. Cancer Res..

[116] Straatman J., Van Der Wielen N., Cuesta M.A., de Lange-de Klerk E.S.M., Jansma E.P., Van Der Peet D.L. (2016). Minimally Invasive Versus Open Total Gastrectomy for Gastric Cancer: A Systematic Review and Meta-Analysis of Short-Term Outcomes and Completeness of Resection: Surgical Techniques in Gastric Cancer. World J. Surg..

[117] Feng Q., Ma H., Qiu J., Du Y., Zhang G., Li P., Wen K., Xie M. (2021). Comparison of Long-Term and Perioperative Outcomes of Robotic Versus Conventional Laparoscopic Gastrectomy for Gastric Cancer: A Systematic Review and Meta-Analysis of PSM and RCT Studies. Front. Oncol..

[118] Aiolfi A., Bona D., Bonitta G., Lombardo F., Manara M., Sozzi A., Schlanger D., Popa C., Cavalli M., Campanelli G. (2024). Long-Term Impact of D2 Lymphadenectomy during Gastrectomy for Cancer: Individual Patient Data Meta-Analysis and Restricted Mean Survival Time Estimation. Cancers.

[119] Chan K.S., Oo A.M. (2024). Learning Curve of Laparoscopic and Robotic Total Gastrectomy: A Systematic Review and Meta-Analysis. Surg. Today.

[120] Seika P., Biebl M., Raakow J., Kröll D., Çetinkaya-Hosgör C., Thuss-Patience P., Maurer M.M., Dobrindt E.M., Pratschke J., Denecke C. (2022). The Learning Curve for Hand-Assisted Laparoscopic Total Gastrectomy in Gastric Cancer Patients. J. Clin. Med..

[121] Brenkman H.J.F., Claassen L., Hannink G., van der Werf L.R., Ruurda J.P., Nieuwenhuizen G.A.P., Luyer M.D.P., Kouwenhoven E.A., van Det M.J., van Berge Henegouwen M.I. (2023). Learning Curve of Laparoscopic Gastrectomy: A Multicenter Study. Ann. Surg..

