# Supplementary material for: Short-Term Outcomes Analysis Comparing Open, Lap-Assisted, Totally Laparoscopic, and Robotic Total Gastrectomy for Gastric Cancer: A Network Meta-Analysis
Source: Cancers (Basel). 2024 Oct 6;16(19):3404. doi: 10.3390/cancers16193404 (PMC11475391; doi:10.3390/cancers16193404)
Supplement: Supplementary file 1 [file cancers-16-03404-s001.zip › Suppl Table S1.pdf]

	Confounding bias	Selection bias	Classification bias	Intervention bias	Missing data bias	Outcome measurement bias	Reporting bias	Overall bias
Eom, 2022Korea [24]	low	moderate	low	serious	serious	moderate	low	serious
Illuminati, 2023 Italy [25]	moderate	moderate	low	low	low	serious	low	serious
Jia, 2023 China [26]	low	moderate	low	low	low	low	low	moderate
Kinoshita, 2022 Japan [27]	low	moderate	low	moderate	low	moderate	low	moderate
Salvador-Roses, 2023 Spain [28]	moderate	moderate	low	moderate	low	serious	low	serious
Zheng, 2023 China [29]	moderate	moderate	low	low	low	moderate	low	moderate
Hu, 2022 China [30]	moderate	moderate	low	low	serious	low	low	serious
Hikage, 2022 Japan [31]	moderate	serious	low	serious	low	moderate	low	serious
Chen, 2022 China [32]	low	low	low	low	low	low	low	low
Cui, 2022 China [33]	moderate	moderate	low	moderate	serious	moderate	low	serious
Di Carlo, 2022 Italy [34]	moderate	moderate	low	low	low	serious	low	serious
Li, 2022 China [35]	low	low	low	low	low	low	low	low
Lin, 2023 China [36]	low	low	low	moderate	serious	moderate	low	serious
Qiu, 2022 China [37]	moderate	moderate	low	low	moderate	moderate	low	moderate
Shibasaki, 2022 Japan [38]	low	low	low	moderate	low	moderate	low	moderate
Wang, 2022 China [39]	low	low	low	low	low	low	low	low
Challine, 2021 France [41]	moderate	moderate	moderate	serious	moderate	serious	low	serious
Fan, 2021 China [42]	low	low	low	low	moderate	low	low	moderate
Feng, 2021 China [43]	low	moderate	low	low	low	moderate	low	moderate
Ko, 2021 Korea [44]	low	moderate	low	low	low	serious	low	serious
Kumamoto, 2022 Japan [45]	moderate	moderate	low	moderate	low	serious	low	serious
Roh, 2021 Korea [46]	low	moderate	low	moderate	low	moderate	low	moderate
Wang, 2021 China [47]	low	low	low	low	low	moderate	low	moderate
Komatsu, 2020 Japan [49]	low	low	low	moderate	low	serious	low	serious
Yang, 2020 China [50]	low	low	low	low	low	moderate	low	moderate
Lee, 2020 Korea [51]	low	moderate	low	moderate	low	moderate	low	moderate
Sakamamoto, 2020 Japan [52]	low	moderate	moderate	serious	low	moderate	low	serious
Zhao, 2019 China [53]	low	low	low	moderate	low	moderate	low	moderate
Ye, 2019 China [54]	moderate	moderate	low	low	low	moderate	low	moderate
Aoyama, 2018 Japan [55]	moderate	moderate	low	moderate	serious	serious	low	serious
Li, 2019 China [56]	low	low	low	low	low	low	low	low
Wang, 2019 China [57]	moderate	moderate	low	low	serious	serious	low	serious
Etoh, 2018 Japan [58]	low	moderate	moderate	serious	low	moderate	low	serious

Chen K, 2017 China [59]	low	low	low	low	low	low	low	low
Chen XZ, 2017 China [60]	low	low	low	low	low	moderate	low	moderate
Lin JX, 2017 China [61]	low	moderate	low	moderate	low	moderate	low	moderate
Kim EY, 2016 Korea [62]	moderate	moderate	low	low	low	low	low	moderate
Kim HB, 2016 Korea [63]	moderate	moderate	low	low	low	serious	low	serious
Wu H, 2016 China [64]	low	moderate	low	moderate	low	moderate	low	moderate
Shu B, 2016 China [65]	moderate	low	low	moderate	low	moderate	low	moderate
Lu Y, 2016 China [66]	moderate	low	low	moderate	low	moderate	low	moderate
Huang, 2017 China [67]	low	moderate	low	low	low	low	low	moderate
Park, 2016 China [68]	low	low	low	moderate	low	serious	low	serious
Shida, 2016 Japan [69]	moderate	moderate	low	moderate	low	moderate	low	moderate
Zhang, 2017 China [70]	moderate	moderate	low	low	low	moderate	low	moderate
Ramagem CAG, 2015 Brazil [71]	moderate	moderate	low	low	low	moderate	low	moderate
Lee, 2015 Korea [72]	moderate	moderate	moderate	moderate	low	serious	low	serious
Lu, 2015 China [73]	low	moderate	low	moderate	low	low	low	moderate
Shen, 2016 China [74]	moderate	moderate	low	serious	low	serious	low	serious
Song, 2015 Korea [75]	moderate	moderate	low	moderate	low	moderate	low	moderate
Lee, 2014 Korea [76]	moderate	moderate	moderate	low	low	serious	low	serious
Lee M, 2013 Korea [77]	moderate	low	low	serious	low	moderate	low	serious
Bo T, 2013 China [78]	low	low	low	low	low	low	low	low
Guan G, 2013 China [79]	moderate	moderate	low	low	low	low	low	moderate
Kim HS, 2013 Korea [80]	moderate	moderate	low	low	low	moderate	low	moderate
Kim KH, 2014 Korea [81]	low	moderate	low	low	low	moderate	low	moderate
Jeong O, 2013 Korea [82]	low	moderate	low	serious	low	low	low	serious
Hong, 2013 China [83]	moderate	low	low	moderate	low	serious	low	serious
Eom BW, 2012 Korea [84]	moderate	moderate	low	moderate	low	serious	low	serious
Kim M, 2011 Korea [85]	moderate	moderate	low	moderate	low	moderate	low	moderate
Yoon, 2012 Korea [86]	moderate	moderate	low	moderate	low	moderate	low	moderate
Sakuramoto S, 2009 Japan [87]	moderate	moderate	low	moderate	low	moderate	low	moderate
Kawamura, 2009 Japan [88]	moderate	moderate	low	low	low	moderate	low	moderate
Mochiki, 2008 Japan [89]	moderate	moderate	low	moderate	low	serious	low	serious
Topal B, 2008 Belgium [90]	moderate	moderate	low	low	low	serious	low	serious
Dulucq, 2005 France [91]	moderate	moderate	low	moderate	low	serious	low	serious

Table S1. Quality assessment of the included studies (ROBINS-I tool). Each domain is evaluated with one of the following: Low, Moderate, Serious, Critical, NI (No Information). The categories of judgement for each study are low, moderate, serious, and critical risk of bias.
