# Supplementary figures and images for: Short-Term Outcomes Analysis Comparing Open, Lap-Assisted, Totally Laparoscopic, and Robotic Total Gastrectomy for Gastric Cancer: A Network Meta-Analysis
Source: Cancers (Basel). 2024 Oct 6;16(19):3404. doi: 10.3390/cancers16193404 (PMC11475391; doi:10.3390/cancers16193404)

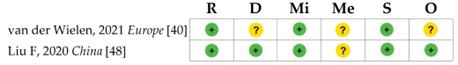

Supplement: Supplementary file 1 [file cancers-16-03404-s001.zip › Suppl Figure S1.tif]
